# Preparation Method of Waterborne Epoxy and the Properties of Its Modified Emulsified Asphalt

**DOI:** 10.3390/ma19214445

**Published:** 2026-10-22

**Authors:** Dewen Su, Jiuran Wen, Jiaqi Sun, Minjia Luo, Yifan Sun, Zuzhong Li, Fang Liu

**Affiliations:** 1 School of Materials Science and Engineering, Chang’an University, Xi’an 710064, China; 2 School of Transportation Engineering, Huaiyin Institute of Technology, Huaian 223001, China

**Keywords:** asphalt pavement, micro-surfacing, binder, modified emulsified asphalt, waterborne epoxy resin

## Abstract

Micro-surfacing is considered as an effective and economical preventive maintenance technique for asphalt pavement. Its performance largely depends on the material compositions of emulsified asphalt binders. To overcome the deficiencies of ordinary micro-surfacing, waterborne epoxy resin emulsified asphalt (WEREA) micro-surfacing can significantly enhance its pavement performance. This work investigated the preparation of waterborne epoxy resin emulsion (WER), solidification behavior, and rheological properties of the solidified WEREA (S-WEREA) by phase inversion method, viscosity, fluorescence microscopy (FM), Fourier-transform infrared (FTIR), and dynamic shear rheometer (DSR) tests, respectively. The results indicated that the optimal preparation conditions of WER were determined as SDS:Span-80 = 5:13 (HLB value 14.2), a 12% emulsifier dosage, a 4712 s^−1^ shear rate, and a 60 °C preparation temperature, based on the evaluation of laser particle size for all specimens. The viscosity, FM, and FTIR tests exhibited that a strong chemical reaction occurred between epoxy resin and curing agent to form abundant crosslinked structures and new functional groups, contributing to improving the high temperature stability of S-WEREA. Dynamic shear rheometer (DSR) tests, including frequency sweep, multiple stress creep and recovery (MSCR), and linear amplitude sweep (LAS), indicated that a 15% WER content could balance the high-temperature deformation resistance and anti-fatigue performance, while excessive WER could adversely affect the low-temperature performance of S-WEREA and also lead to macroscopic phase separation at high-temperature. This study is expected to lay an experimental foundation for the wide application of WEREA in micro-surfacing.

## 1. Introduction

Micro-surfacing is an effective preventive maintenance technology, widely used in asphalt pavements, due to its fast construction, low energy consumption and cost savings [1]. However, in practical applications, micro-surfacing still faces several technical challenges, such as rutting, abrasion, ravelling and so on, mainly attributed to the inadequate stability and weak bonding strength of emulsified asphalt binders [2,3]. To address these problems, suitable modification methods need to be developed to enhance binder performance.

Many researchers have initiated investigating complex multiphase emulsified asphalts for micro-surfacing mixtures with various modifiers, including waterborne epoxy resin (WER), styrene-butadiene rubber (SBR), polyurethane (PU), styrene-butadiene-styrene block copolymer (SBS) and so on [4,5,6]. Among them, WER has exhibited excellent bonding, good thermal stability and aging resistance, which emerged as a promising binder and aroused extensive interest from road-related researchers. Wu et al. highlighted that WER-modified emulsified asphalt could significantly enhance elastic components, creep-recovery, fatigue life, and fracture energy [7]. Also, Liu et al. demonstrated that waterborne epoxy resin-SBR compound modified emulsified Asphalt (WER-SCMEA) could possess good pavement performance and greatly alleviate the damages in the condition of long-term water immersion and freeze–thaw cycles [8]. WER could undergo polymerization reactions in emulsified asphalt and significantly improve the high-temperature elastic recovery performance of emulsified asphalt by mainly physical modification. However, excessive WER had certain adverse effects on the low-temperature performance of WEREA [9].

Currently, most studies on WER-modified micro-surfacing have focused on optimizing the mixing ratios of raw materials, testing the storage stability and dispersion stability of WER, evaluating its pavement performance, and discussing the modification mechanism [10,11]. In particular, the WER content strongly influences the performance of the resulting binder and mixture. Yang et al. reported that the cured WER formed a continuous skeleton in the emulsified asphalt at a critical content of about 15%, above which the elastic recovery exceeded 98% and the high-temperature deformation resistance increased markedly, while the low-temperature performance deteriorated and the content had to be strictly controlled [12]. Chen et al. further showed that a higher WER content increased the complex shear modulus, decreased the phase angle and shifted the MSCR response from viscoelastic to elastic, thereby improving the deformation, rutting and fatigue resistance [13]. However, systematic research on the material compositions and preparation method of WER remains insufficient. Therefore, focusing on WER used in emulsified asphalt, this study selected the raw materials, developed the emulsifier formulation, and analyzed the effects of the emulsifier and preparation method on WER particle size using a laser particle size analyzer. The curing behavior of WEREA was then investigated using a Brookfield viscometer. Subsequently, the phase morphology of S-WEREA was observed using fluorescence microscopy (FM), and its chemical functional groups were analyzed by Fourier-transform infrared spectroscopy (FTIR). Finally, the rheological properties of S-WEREA were evaluated through frequency sweep, multiple stress creep and recovery (MSCR), and linear amplitude sweep (LAS) tests using a dynamic shear rheometer (DSR), and the results of all tests were analyzed comprehensively to determine the optimal WER content for micro-surfacing binders.

## 2. Materials and Experiments

### 2.1. Material Details

#### 2.1.1. SBR Emulsified Asphalt

This work selected SBR modified emulsified asphalt as elementary binder, which was prepared in the laboratory. Its fundamental technical properties, including the residue on sieve, content of evaporation residue, storage stability and properties of evaporation residue, are detailed in Table 1, respectively.

#### 2.1.2. Resins and Curing Agent

In this work, epoxy resin (E-51) was purchased from Nantong Xingchen Synthetic Materials Co., Ltd. (Nantong, China) and curing agent (CYDHD-220) was purchased from Guangzhou Qian Yiyuan Synthetic Material Technology Co., Ltd. (Guangzhou, China), both regarded as common industrialized products, were employed. Their technical properties are shown in Table 2 and Table 3.

#### 2.1.3. Formulation of Emulsifier

Generally, the efficiency of emulsifiers was evaluated by the hydrophilic–lipophilic balance value (HLB), defined by Equation (1) [18].

(1) $$\begin{array}{c} \mathrm{HLB}=7+\sum \left(\mathrm{hydrophilic group numbers}\right)+\sum \left(\mathrm{lipophilic group numbers}\right) \end{array}$$

Different emulsifiers possess various hydrophilic–lipophilic balance (HLB) values due to their numbers of hydrophilic and lipophilic groups. An HLB value between 8 and 18 is suitable for an oil-in-water emulsion [19]. In contrast to a single emulsifier, the synergistic effect of composite emulsifiers is conductive to the emulsification of epoxy resin [20]. Therefore, in this work, SDS emulsifier (A) and Span-80 emulsifier (B), purchased from Xi’an Jingbo Biotechnology Co., Ltd. (Xi’an, China), were selected, exhibiting 40 and 4.3 HLB values, respectively. The comprehensive HLB value of both emulsifiers was calculated according to Equation (2):

(2) $$\begin{array}{c} \mathrm{HLB}=\frac{m_{A}\times {\mathrm{HLB}}_{A}+m_{B}\times {\mathrm{HLB}}_{B}}{m_{A}+m_{B}} \end{array}$$

where m_A_ and m_B_ represent the mass of SDS and Span-80, respectively; HLB_A_ and HLB_B_ represent the HLB values of SDS and Span-80, respectively.

Based on the required HLB range of 8–18 for emulsifying E51 resins, the composite emulsifiers with comprehensive HLB values were formulated by different mass ratios to achieve the optimal effect of emulsification for the waterborne epoxy resin emulsion. The basic properties of SDS and Span-80 are shown in Table 4, while the various blending ratios for the composite formulations are shown in Table 5.

### 2.2. Preparation of WER

**Figure 1:**
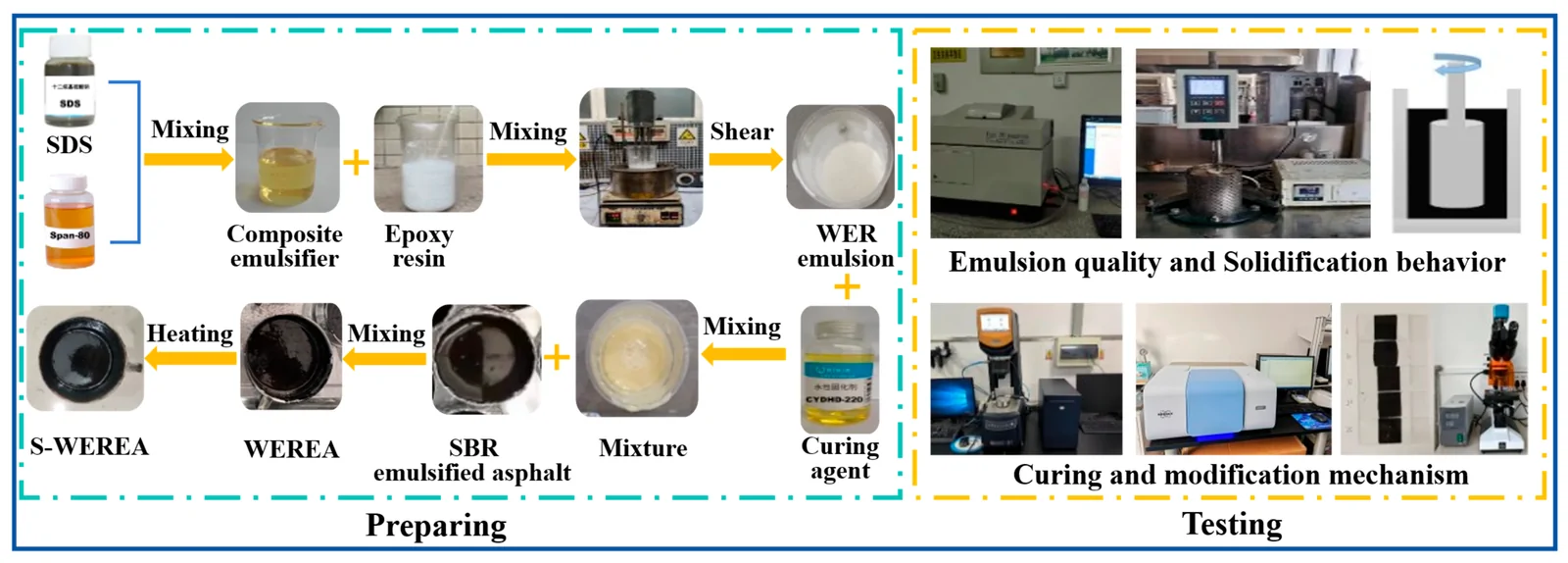
Flowchart of experiment design.

**Figure A1:**
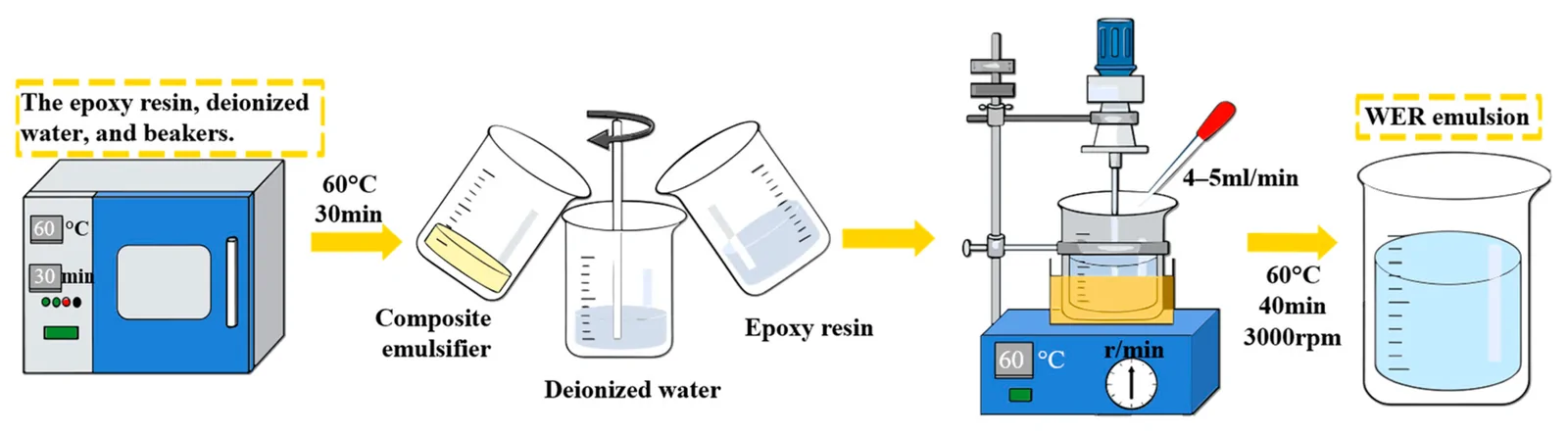
The preparation process of WER.

In this work, the phase inversion method was employed to prepare waterborne epoxy resin emulsion with the solid content of approximately 55%. Specifically, 300 g of epoxy resin (E51) was used in each batch, the composite emulsifier was added at 3, 6, 9, 12, 15 and 18% (9, 18, 27, 36, 45 and 54 g, respectively), and the amount of deionized water was adjusted to 254, 261, 268, 276, 283 and 290 g, respectively. The preparation process was conducted in two steps (Figure 1 and Figure A1).

In the first step, in batches, epoxy resin, deionized water, and beakers were preheated in an oven (101-3S, Shanghai Lichen Instrument Technology Co., Ltd., Shanghai, China) at temperature (40, 50, 60, 70 and 80 degrees C). A small amount of deionized water was used to completely mix the two emulsifiers. Subsequently, the composite emulsifier was slowly poured into the beaker, followed by the epoxy resin. Throughout this process, a glass rod was employed for manual stirring to ensure a homogeneous mixing. Then, in batches, the blended solutions were carefully transferred into a temperature-controlled oil bath at temperature (40, 50, 60, 70 and 80 degrees C).

In the second step, the solutions were sheared at rotational speeds of 2000, 2500, 3000, 3500 and 4000 r/min using a dedicated shear mixer (JRJ300-S; Shanghai Jingqi Instrument Co., Ltd., Shanghai, China; rotational speed: 200~11,000 r/min; output power: 300 W; rated output torque: 0.3 N∙m; stirring-head diameter: Φ60 mm; rotor–stator clearance: 2 mm; dimensions: 250 × 350 × 720 mm), and the corresponding shear rates were 3141, 3926, 4712, 5497 and 6283 s^−1^, respectively, as calculated from Equation (A1). Simultaneously, the preheated deionized water was added dropwise using a dropper at a controlled rate of 4–5 mL/min. Upon phase inversion, which was indicated by a continuous decrease in viscosity, the remaining deionized water was added to the solution, and the mixture was further sheared for 30 min. After cooling to room temperature, the emulsion was bottled and stored in a light-proof environment.

### 2.3. Preparation of WEREA

**Figure A2:**
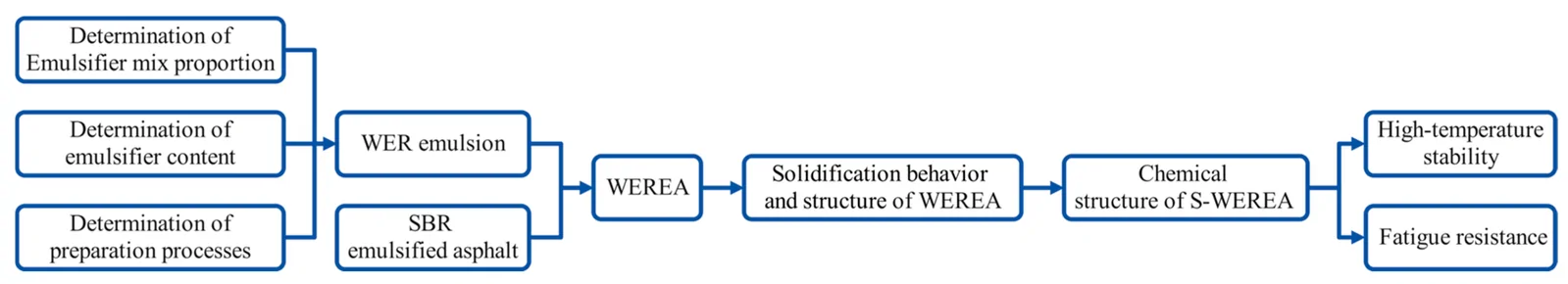
The flowchart of the research methodology.

Firstly, the prepared WER and curing agent were mixed and stirred continuously to form a uniform solution. Subsequently, the solution was added into SBR emulsified asphalt. Finally, the evaporated residue from the above-mentioned solution was obtained by following steps (Figure 1 and Figure A2):

(1) The mixed solution was kept in the drying oven at 70 °C for 6 h and stirred every half hour to prevent the formation of asphalt film on the surface, ensuring continuous moisture evaporation.
(2) Once SBR emulsified asphalt began to demulsify, the oven temperature was increased to 110 °C and the mixed solution was stirred every 15 min.
(3) When asphalt temperature rose to 110 °C, the moisture was fully evaporated, and the residue preparation was completed. The entire process took approximately 60 min.

### 2.4. Test Methods

#### 2.4.1. Laser Particle Size Test

The uniformity and stability of the WER system depend on the particle size distribution of WER emulsion, which also reflect the emulsifying efficiency of the emulsifier and the preparation quality of WER emulsion. In this work, firstly, the Winner2000E laser particle size analyzer (Jinan Micro-Nano Technology Co., Ltd., Jinan, China) was employed to measure the particle size distribution of WER with different composite emulsifiers and then confirm the optimal mass ratio of composite emulsifier system. Secondly, E51 resin was emulsified with the optimal composite emulsifiers. The mass fractions of these emulsifiers, relative to E51 resin, were 3%, 6%, 9%, 12%, 15%, and 18%, respectively. The WER emulsions were also evaluated by laser particle size analysis to obtain the reasonable mix proportion of the emulsifier and E51 resin. Finally, under different shear rates (3141, 3926, 4712, 5497, and 6283 s^−1^) and temperatures (40, 50, 60, 70, and 80 °C), E51 resins using the reasonable mix proportion were emulsified and their particle size distributions were analyzed, respectively.

#### 2.4.2. Viscosity Test

Due to the curing behavior of WER system and the demulsification of emulsified asphalt under normal temperature, the viscosity of WEREA continuously increases, significantly affecting the workability of WEREA mixtures. In this work, the Brookfield viscometer (DV-II + Pro, Brookfield Engineering Laboratories, Inc., Middleboro, MA, USA) was employed to test the time-dependent viscosity curves of WEREA at 25 °C with varying amounts of WER. Each sample was independently prepared and measured three times.

#### 2.4.3. Fluorescence Microscope

To further investigate the interaction between WER and emulsified asphalt, as well as the effect of different WER dosages on the structure of modified emulsified asphalt, the evaporated residues of WEREA at various dosages were observed using the fluorescence microscope (ZEISS Axio Imager.Z2, Carl Zeiss Optical Co., Ltd., Jena, Germany). The images were acquired at the same magnification, and multiple fields of view were analyzed for each specimen.

#### 2.4.4. FTIR

In this work, Fourier-transform infrared (Bruker TENSOR II, Bruker (Beijing) Scientific Technology Co., Ltd., Beijing, China) spectrometer equipped with attenuated total reflection (ATR) was used to conduct functional group analysis of the E51 resin, curing agent, and cured products. The infrared spectra were recorded with the resolution of 4 cm^−1^, covering the wavenumber range between 400 and 4000 cm^−1^.

#### 2.4.5. DSR

In the Strategic Highway Research Program (SHRP), most rheological indexes were established based on the assumption of linear viscoelasticity. To evaluate evaporated residues using SHRP rheological indices, it is essential that the test materials must fall within the linear viscoelastic region (LVE). In this study, the LVE was defined as being within 4.6%, which meets the requirements for subsequent tests.

1. Frequency sweep

The rheological properties of the WEREA evaporated residues were characterized through frequency sweep tests within a temperature range of 46–76 °C (at 6 °C increments) to assess their performance under conditions simulating varying vehicle speeds. Using a 25 mm steel parallel plate, all measurements were conducted within the linear viscoelastic (LVE) region, determined by an amplitude sweep, at a fixed strain of 1% over a frequency range of 0.1 to 100 rad/s.

2. MSCR

This study, following the ASTM D7405 standard [21], performed continuous “loading–unloading” cycle tests on residues with varying WER content at the test temperature of 64 °C, under two stress levels (20 cycles at a 0.1 kPa stress level and 10 cycles at a 3.2 kPa stress level), with the first 10 cycles used for specimen adjustment and excluded from the calculation of performance metrics.

3. LAS

The LAS test, based on the viscoelastic continuum damage (VECD) theory, was used to predict the fatigue damage of asphalt materials, allowing for the rapid and accurate assessment of their fatigue performance under certain strain level. In this study, the LAS test was conducted in accordance with AASHTO TP-101 to evaluate the fatigue performance of WEREA residues.

The residue specimens were subjected to the aging treatment in the rolling thin film oven test (LBH-85, Hebei Dahong Experimental Instrument Co., Ltd., Cangzhou, China). The LAS test, utilizing 8 mm stainless steel parallel plates with the gap of 2 mm at 25 °C, consists of two phases: the first phase involves a frequency sweep test at a controlled strain level of 0.1%, covering frequency range of 0.2 to 30 Hz, aimed at determining the linear viscoelastic properties of specimens. The second phase is strain sweep test with an increasing strain, conducted at loading frequency of 10 Hz, the duration of 310 s, and loading amplitude ranging from 0.1% to 30%. Each sample was independently prepared and measured three times.

## 3. Results and Discussion

### 3.1. The Influence of Emulsifier Mix Proportion on Particle Sizes of WER

**Figure 2:**
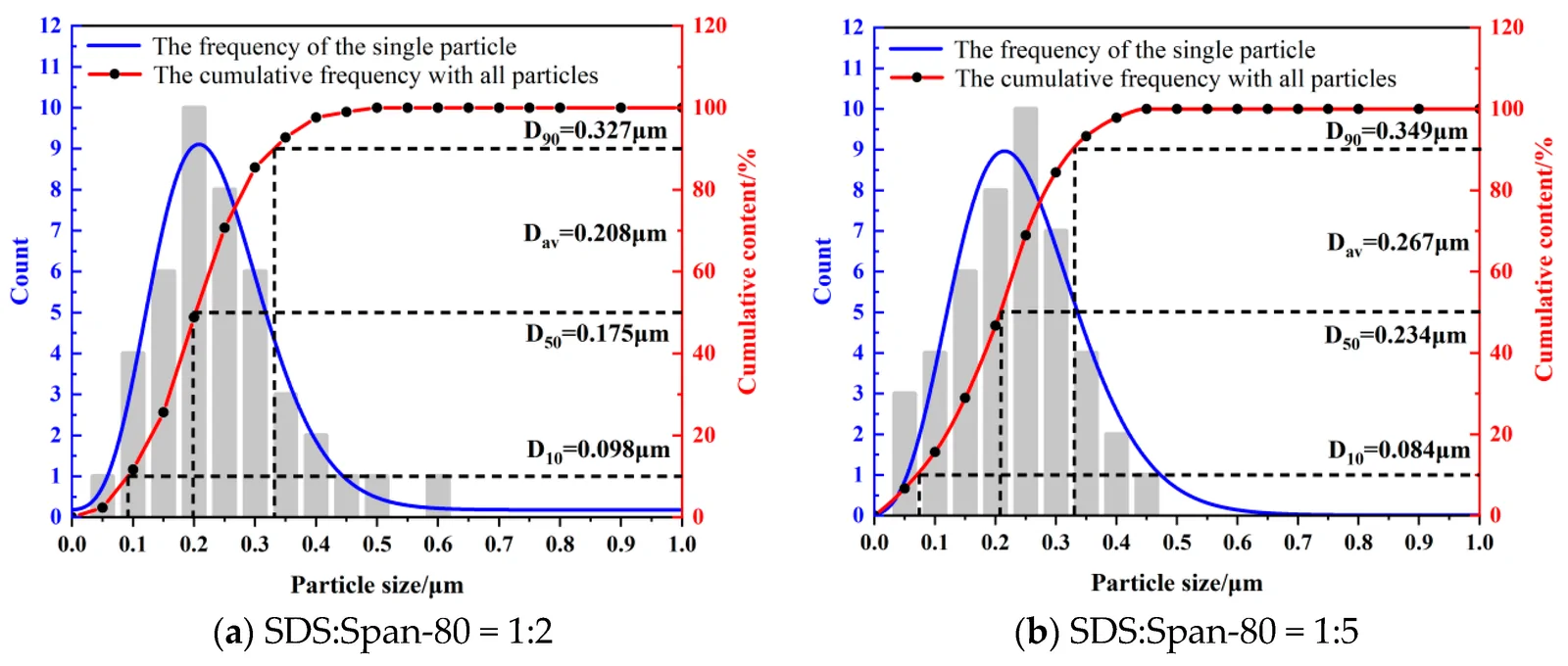

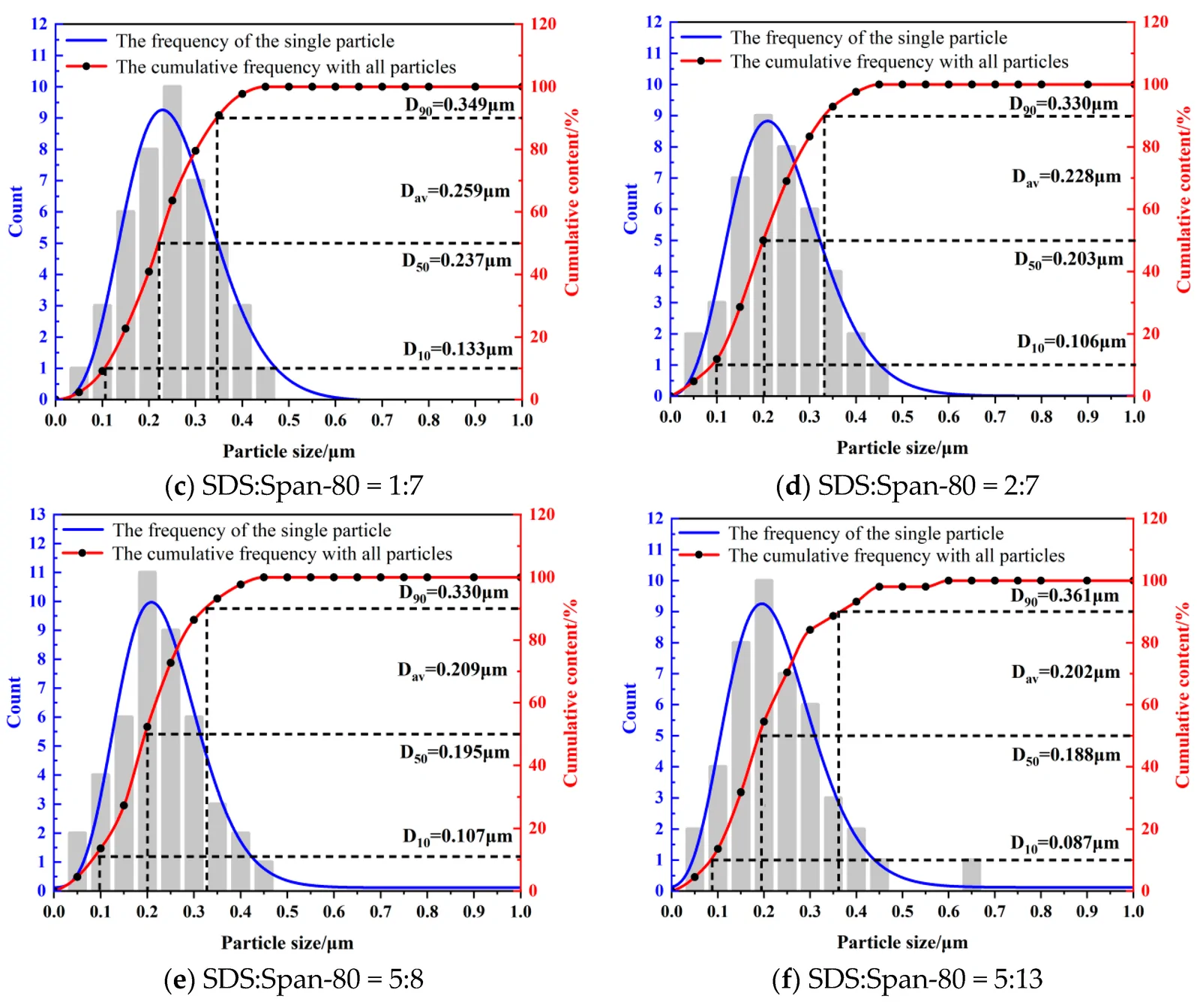
Particle size distribution in waterborne epoxy resins prepared with emulsifiers of different compounding ratios.

Figure 2 shows the particle size distribution results of WER prepared with different mix proportions of composite emulsifier at room temperature. The *x*-axis denotes the particle size, while the left *y*-axis denotes the frequency of certain particle size and the right *y*-axis denotes the cumulative frequency till current particle size.

In this test, D_10_, D_50_, and D_90_ represent the particle sizes corresponding to cumulative particle size distributions reaching 10%, 50%, and 90% of the total volume, respectively, while D_av_ denotes the average particle size of sample. Moreover, a function fitting method was applied to analyze the variation trend of the particle size distribution and it was found that the Giddings distribution function (Equations (3) and (4)) showed a superior fit due to its excellent adaptability to data with peak value fluctuations.

(3) $$y=\frac{A}{\omega }\sqrt{\frac{x_{c}}{x}}{\text{bessel\_I}}_{1}\left(\frac{2\sqrt{x_{c}x}}{\omega }e^{\frac{-x-x_{c}}{\omega }}\right)$$

(4) $${\mathrm{beseel}}_{-}I_{1}=\frac{x}{2}+\frac{x^{3}}{2^{2}\times 4}+\frac{x^{5}}{2^{2}\times 4^{2}\times 6}+\cdots$$

where A is the area coefficient, ω is the width coefficient, x_c_ is the center coefficient, and beseel_−_I_1_ is the Bessel function.

As shown in Figure 2, the particle size is mainly within the range of 0.1 μm to 0.4 μm. Generally speaking, an HLB value of 8–18 is suitable for an oil-in-water emulsion [19]. In this work, although the HLB values of composite emulsifiers were between 8 and 18, the particle sizes of WER exhibited significant differences. When epoxy resin was emulsified using a single emulsifier, the molecular packing density of the emulsifier was reduced due to the steric hindrance of the hydrophilic head groups [22]. Nevertheless, driven by the synergistic action of SDS and Span-80, SDS was apt to be closer to water molecules, while Span-80 was nearer to oil molecules. This arrangement allowed the molecules to contact more tightly, increased the packing density and thereby enhanced interfacial stability, resulting in superior emulsification effect compared to using a single emulsifier.

When the D_av_ of WER reached its minimum value (0.202 μm), the HLB value of the composite emulsifier was 14.2. At this point, the error in the Giddings distribution function was also at the lowest, indicating that the emulsifier mass ratio of 5:13 for SDS and Span-80 exhibited the optimal emulsification effect.

### 3.2. The Influence of Emulsifier Content on Particle Sizes of WER

**Figure 3:**
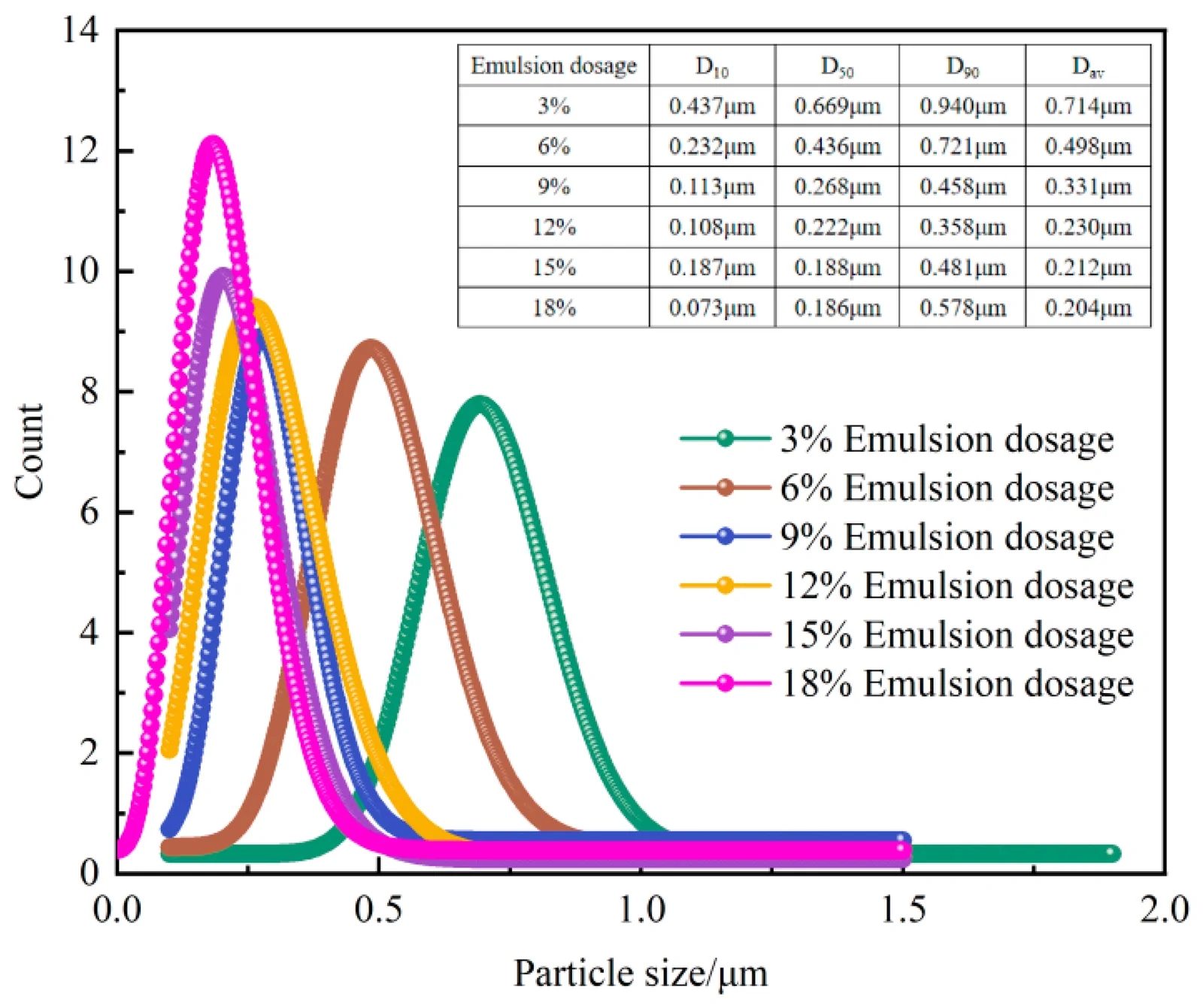
Particle size distribution of WER at different emulsion dosages.

Under the above-mentioned optimal emulsifier ratio of 5:13 (SDS:Span-80), epoxy resins were emulsified by the emulsifiers added at mass ratio of 3%, 6%, 9%, 12%, 15% and 18%, respectively. The particle size results of WER are presented in Figure 3.

It can be seen from Figure 3 that with the increase in the amount of the compound emulsifier, the average particle size of WER gradually decreased, the particle size distribution became denser. When the composite emulsifier content changed from 3% to 9%, the average particle size of the WER varied from 0.714 μm to 0.331 μm, implying that the lower the addition of composite emulsifier, the larger the particle size of WER. This can be attributed to the fact that at a low emulsifier dosage the hydrophilic segments in WER were relatively abundant, which prevented the emulsifier molecules from adsorbing water and mixing uniformly in the emulsion. Hence, the water membrane containing composite emulsifier could not completely encapsulate the microparticles of resin and weaken the formation of effective interfacial film between water molecule and epoxy resin.

When the composite emulsifier content was from 12% to 18%, the average particle size of WER changed from 0.230 μm to 0.204 μm. Within this range, as the emulsifier content further increased, the water membrane containing composite emulsifier gradually coalesced to slow down the surface tension of the emulsifier and fully covered the microparticles of resin [23], which resulted in an enhanced adsorption capacity of the emulsifier molecules on the epoxy resin particles and formed the steadier interfacial film on the surface of the epoxy resin particles.

Apparently, although within the experimental content range, the average particle size of the emulsion consistently decreases with the increase in emulsifier content, it was observed that within the dosage range of 3% to 9%, variations in the emulsifier content exerted a marked effect on the average particle size, leading to a reduction of approximate 53.6%. While, once the emulsifier content belonged to the range of 12% to 18%, a reduction of approximate 11.3% for the average particle size only occurred. Therefore, when the epoxy resin was sufficiently encapsulated by the emulsifier on the content of 12%, the particle size no longer decreased appreciably and the interface could be nearly saturated. In addition, excess emulsifier would cause an adverse influence on the long-term service performance of micro-surfacing mixture [24,25]. In conclusion, it is advisable to select the emulsifier content of 12% during the preparation process of WER.

### 3.3. The Influence of Preparation Processes on Particle Sizes of WER

#### 3.3.1. Shear Rate

**Figure 4:**
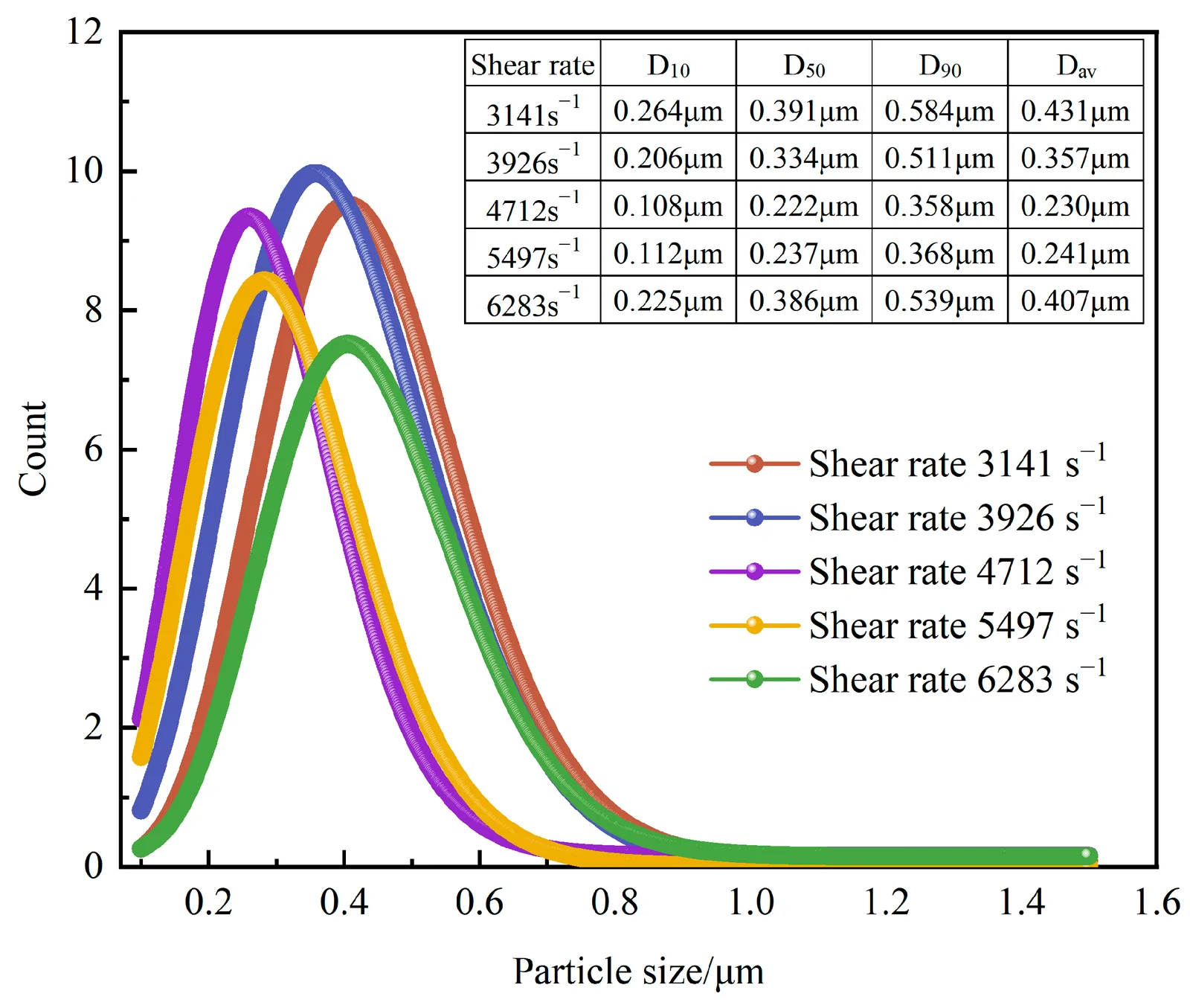
Particle size distribution of WER at different shear rate.

As illustrated in Figure 4, with the rise in the shear rate, the average particle size of the emulsion decreased first and then increased. No matter whether the shear rate is high or low, the emulsions exhibited a broader particle size distribution and a greater average particle size.

During the shear process, epoxy resin particles were dispersed in water, leading to the expansion of water–oil interface. Concurrently, the flat interface turned into a curved surface. As indicated by Equation (5), the curvature of interface generated additional pressure, requiring the shear device to supply sufficient energy to compensate the extra energy for the formation of the curved liquid surface.

At shear rates of 3141 s^−1^ and 3926 s^−1^, the shear strain provided by the stirring blades was insufficient and strain energy was inadequate to achieve complete phase transition in WER, reducing the efficiency of epoxy resin dispersion and emulsification. However, at higher shear rates, such as 5497 s^−1^ and 6283 s^−1^, the collision frequency of emulsion droplet increased, accelerating the aggregation of droplet and causing the formation of larger dispersed particles [26]. Considering the average particle sizes of emulsions prepared at various shear rates, it was concluded that WER produced at the shear rate of 4712 s^−1^ achieved the optimal particle size.

(5) $$∆p=\gamma \left(\frac{1}{R_{1}}+\frac{1}{R_{2}}\right)$$

where ∆p is the additional pressure, γ is the interfacial tension coefficient of the curved liquid surface, and *R*_1_ and *R*_2_ are the two orthogonal radii of curvature of the point on the curved surface. When the curved surface is a spherical surface, *R*_1_ = *R*_2_ = *R*, $∆p=\frac{2\gamma }{R}$.

#### 3.3.2. Preparation Temperature

**Figure 5:**
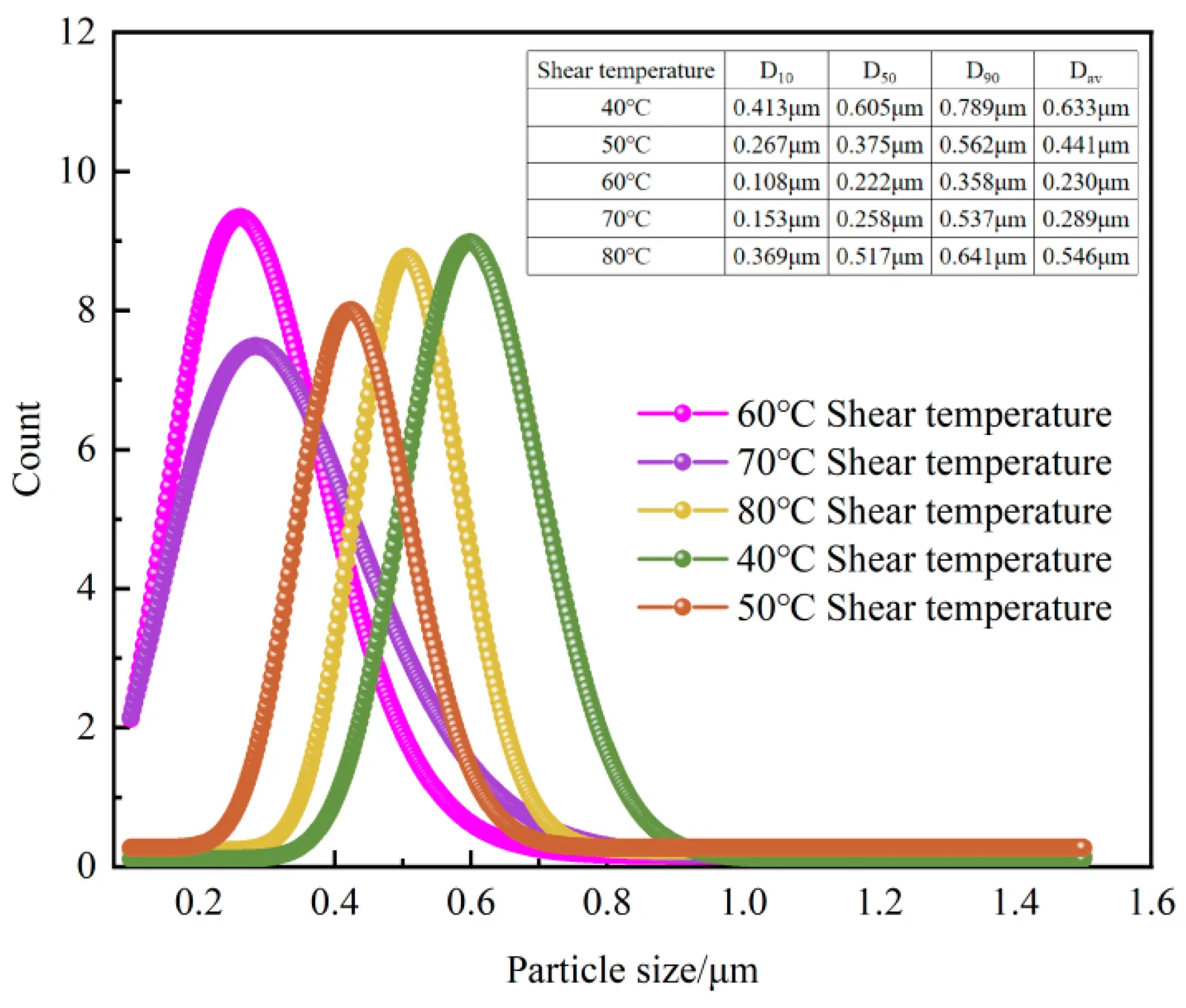
Particle size distribution of WER at different preparation temperatures.

As shown in Figure 5, within the temperature range of 40 °C to 60 °C, the increase in temperature facilitated the preparation of a stable WER, reducing the viscosity of the epoxy resin and decreasing the possibility of entanglement among emulsion particles, and thereby minimized the particle size.

However, when the temperature exceeded 60 °C, the further increases in temperature were no longer conducive to the reduction in particle size. This phenomenon stemmed from the fact that when the preparation temperature became too high, the kinetic energy possessed by the dispersed phase ions increased, causing a large number of molecules in emulsion to be excessively active. Moreover, as the preparation temperature continuously rose, the possibility of collisions between emulsion particles increased, while the binding force between the lipophilic groups in emulsifier and the epoxy resin, as well as the hydrogen bond energy between the hydrophilic groups and water, decrease simultaneously. These reasons resulted in smaller droplets merging into larger ones, which increased the average droplet size and influenced the distribution width of particle size and the stability of interfacial film between emulsifier and epoxy resin [27]. At 60 °C, WER could achieve the smallest average particle size (0.230 μm) and the stabler interfacial film formed by the composite emulsifier. Therefore, it was eventually determined that the optimal preparation temperature of WER was 60 °C.

In summary, the particle size distribution was exhibited clearly in Figure 2, Figure 3, Figure 4 and Figure 5 and the average particle size corresponds to the region of the dominant particle distribution. Bridging average particle size, particle size distribution, and storage stability, the optimal preparation condition for WER was determined as HLB value 14.2, 12% emulsifier dosage, a 4712 s^−1^ shear rate, and a preparation temperature of 60 °C.

### 3.4. Solidification Behavior of WEREA

The modification mechanism of waterborne epoxy resin in emulsified asphalt is that curing reaction occurs between epoxy resin and curing agent and the cured product is physically blended with demulsified asphalt to form an interlocked structure, which is a more compact and stable three-dimensional structure. In this work, solidification behavior of WEREA was evaluated by rotational viscometry (RV) tests at room temperature.

**Figure 6:**
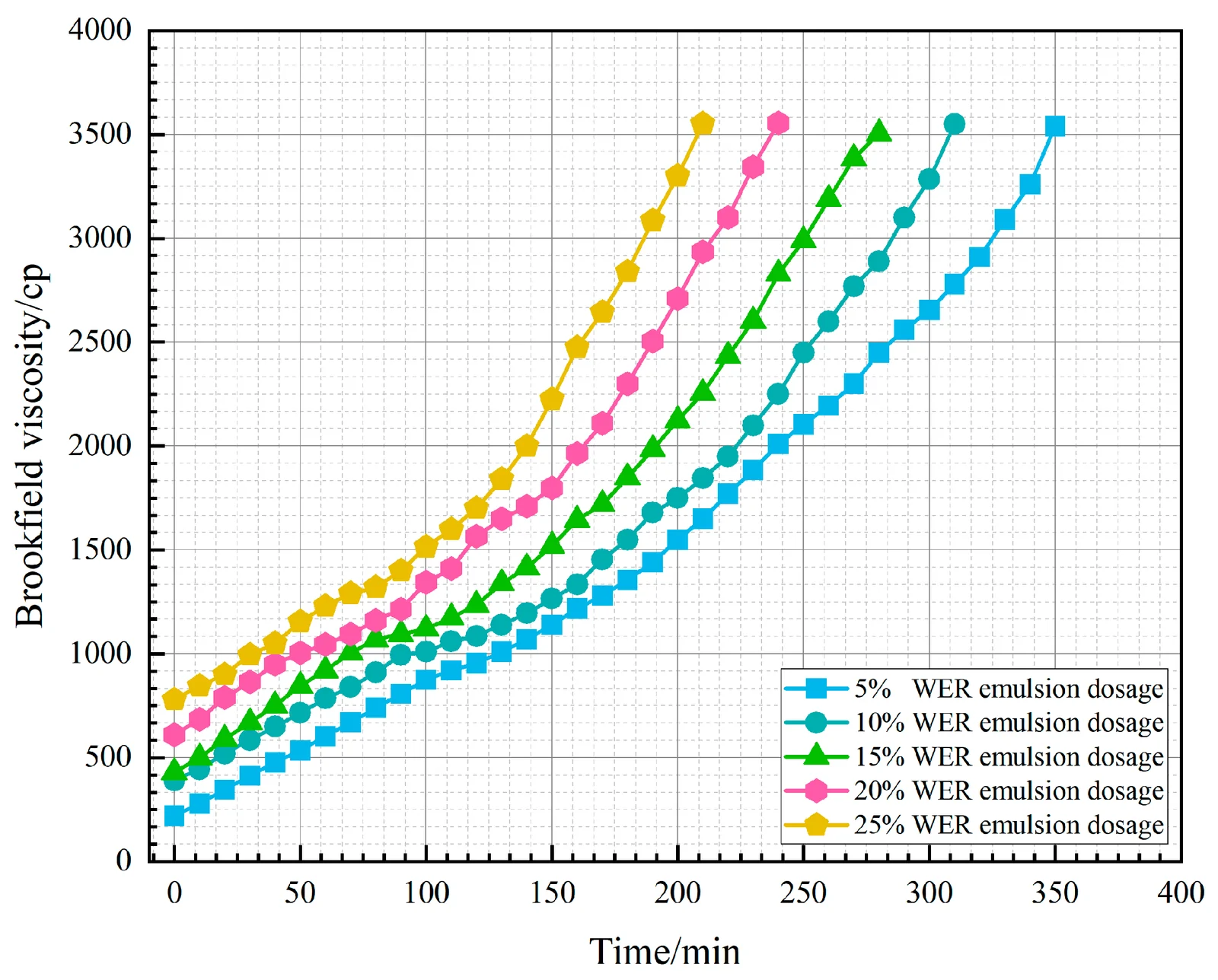
Viscosity–time curves of varying WER emulsion dosage emulsified asphalt.

As can be seen in Figure 6, with the extension of testing time, the viscosity of WEREA gradually increased. Similarly, as the content of waterborne epoxy resin increased, the viscosity of WEREA also rose. After the emulsions were mixed together, both the emulsified asphalt and WER underwent demulsification and the reaction between epoxy resin and curing agent was in the initial stage, leading to lower viscosity in WEREA. Subsequently, the cured product from WER gradually grew and the action of cross linking slowly appeared, enhancing the viscosity of WEREA. Thus, it could be seen that the degree of curing was positively correlated with viscosity. Ultimately, as the curing reaction proceeded and the emulsified asphalt demulsified, the viscosity of WEREA gradually approached a stable value.

As for the influence of WER content on the curing process, when the addition of WER varied from 5% to 25%, the viscosity of WEREA exhibited a growing trend. Apparently, after about 100 min, the more the dosage of WER, the faster the increase in the viscosity. Considering the feasibility of the construction and the modification effect of WER, there was a reasonable value of the WER addition.

### 3.5. Fluorescence Image of S-WEREA

**Figure 7:**
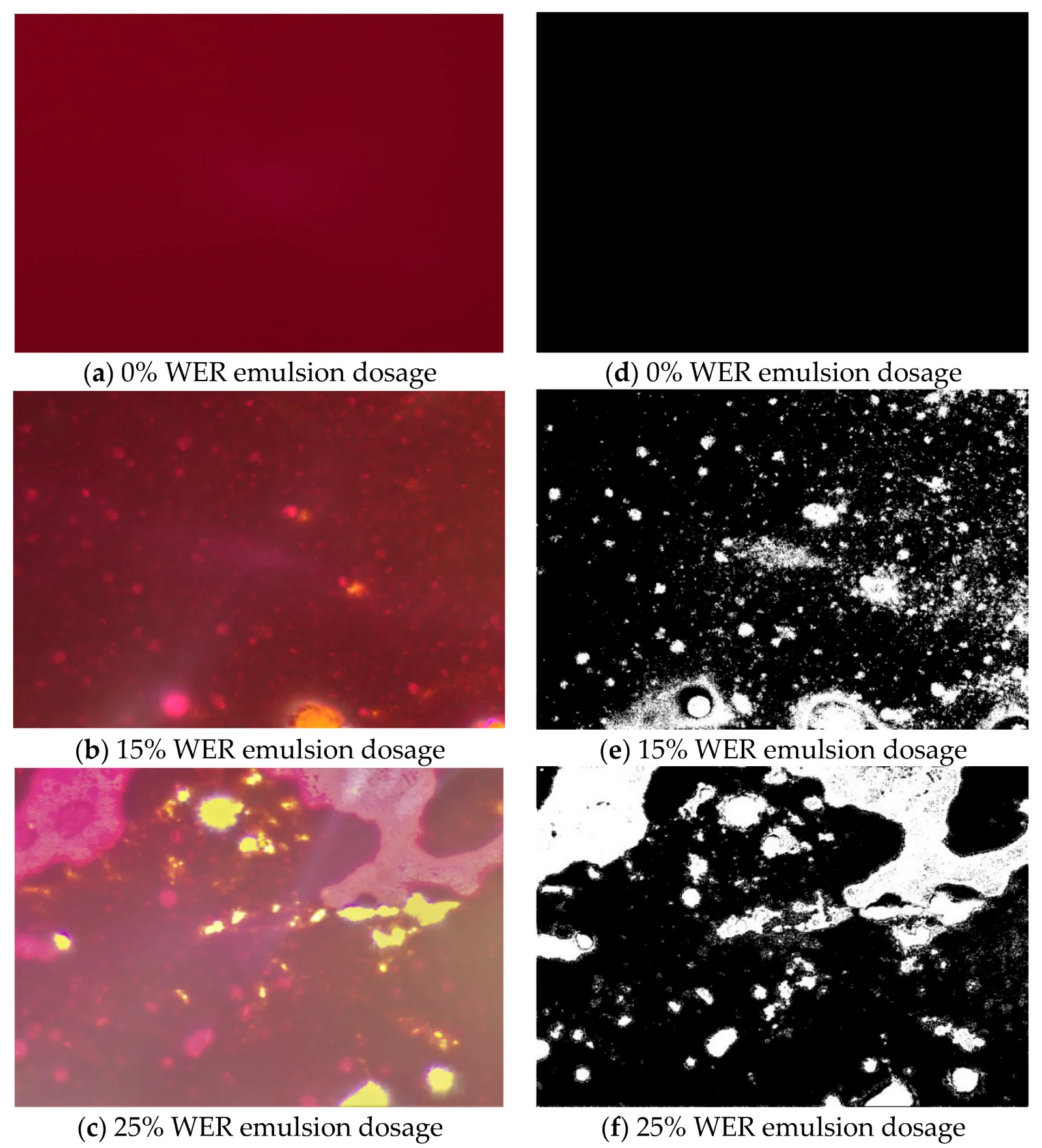
Fluorescence microscopy images of emulsified asphalt with 0%, 15%, and 25% WER dosages (**a**–**c**); corresponding binarization results (**d**–**f**).

**Figure A4:**
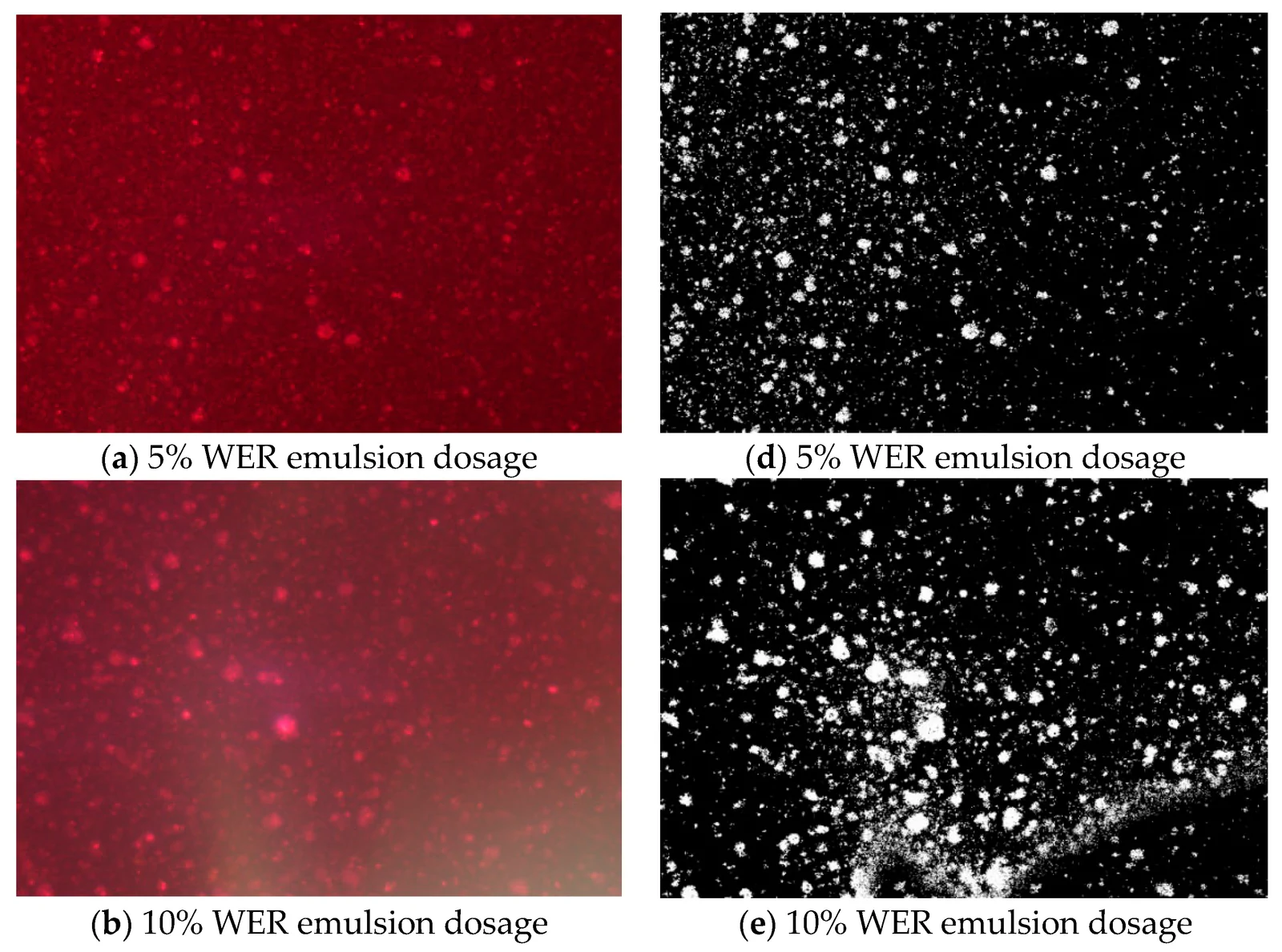

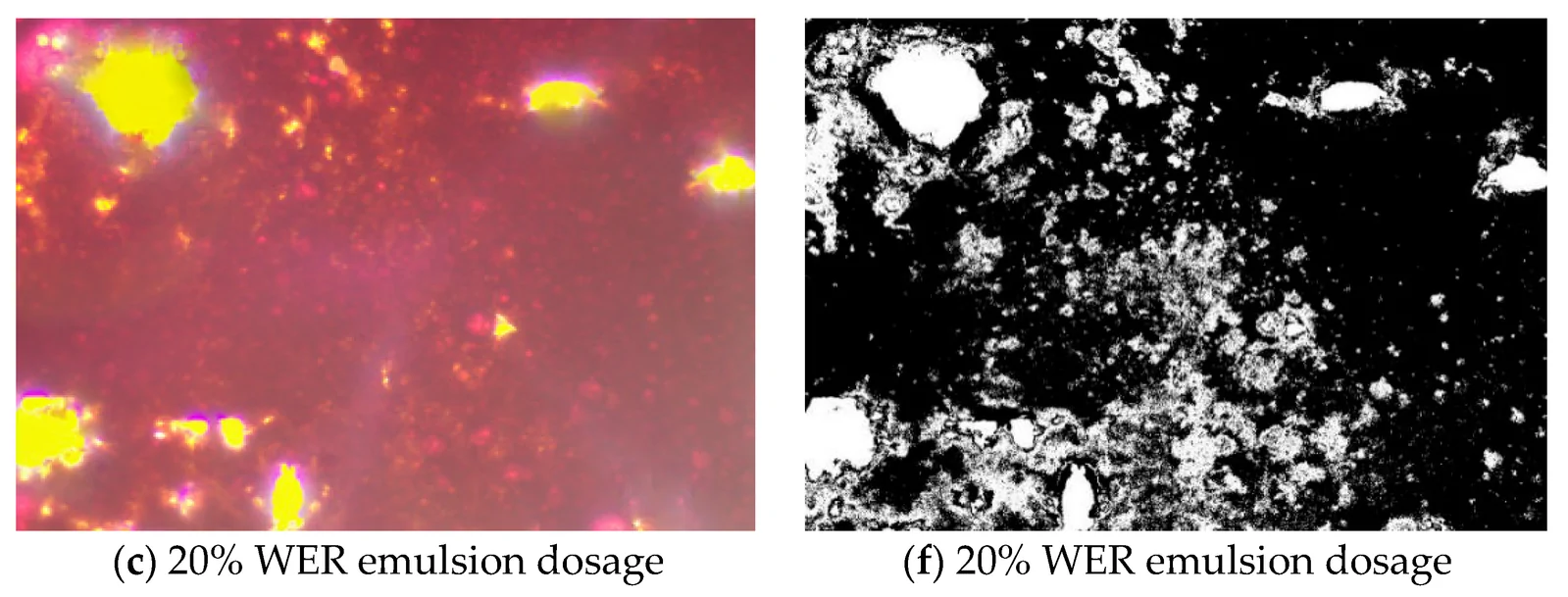
Fluorescence microscopy images of emulsified asphalt with 5%, 10%, and 20% WER dosages (**a**–**c**); corresponding binarization results (**d**–**f**).

In Figure 7a–c and Figure A4a–c, the fluorescent spots were mainly from the cured product of WER, while the non-fluorescent parts were regarded as demulsified asphalt. When the content of WER was low, epoxy resin spots were small and evenly dispersed in asphalt phase, forming a blurry sea island structure. When the content of WER reached 15%, a few large aggregations appeared. With the further increase in the dosage, more cured product of WER gathered, the growing large islands began to coalesce with each other, and then formed a few irregular spots.

To semi-quantitatively analyze the dispersion of the WER cured product in WEREA, the fluorescence images were acquired at the same magnification and field of view, and the Image ProPlus (IPP) 6.0 software (Media Cybernetics, Inc., Rockville, MD, USA) was employed to convert them into binarized images. Filtering and denoising were then applied to improve the image quality. As shown in Figure 7d–f and A4d–f, the white part of the binary images represented the cured product of WER, and the black part was SBR-modified asphalt. The binarized images were segmented to estimate the area fraction of the WER cured product in WEREA. The parameter R was defined as the area ratio of the white part to the whole image, which was calculated based on Equation (6); R was averaged over the available fields of view for each specimen. The results are shown in Table 6. As the amount of WER increased, the area of the fluorescent parts gradually increased, and R reached 13.8% at the WER content of 15%. It should be noted that R is only a semi-quantitative estimate of the fluorescent area fraction; no metrics of domain size, connectivity, or heterogeneity were quantified, and no numerical correlation between R and the rheological results is reported.

(6) $$R=\frac{\sum_{i=1}^{n}i}{\sum_{j=1}^{m}j}\times 100\%$$

where *i* represents the fluorescent pixel points of the waterborne epoxy resin, and *j* represents all the pixel points in the image.

### 3.6. Structural Characterization

**Figure 8:**
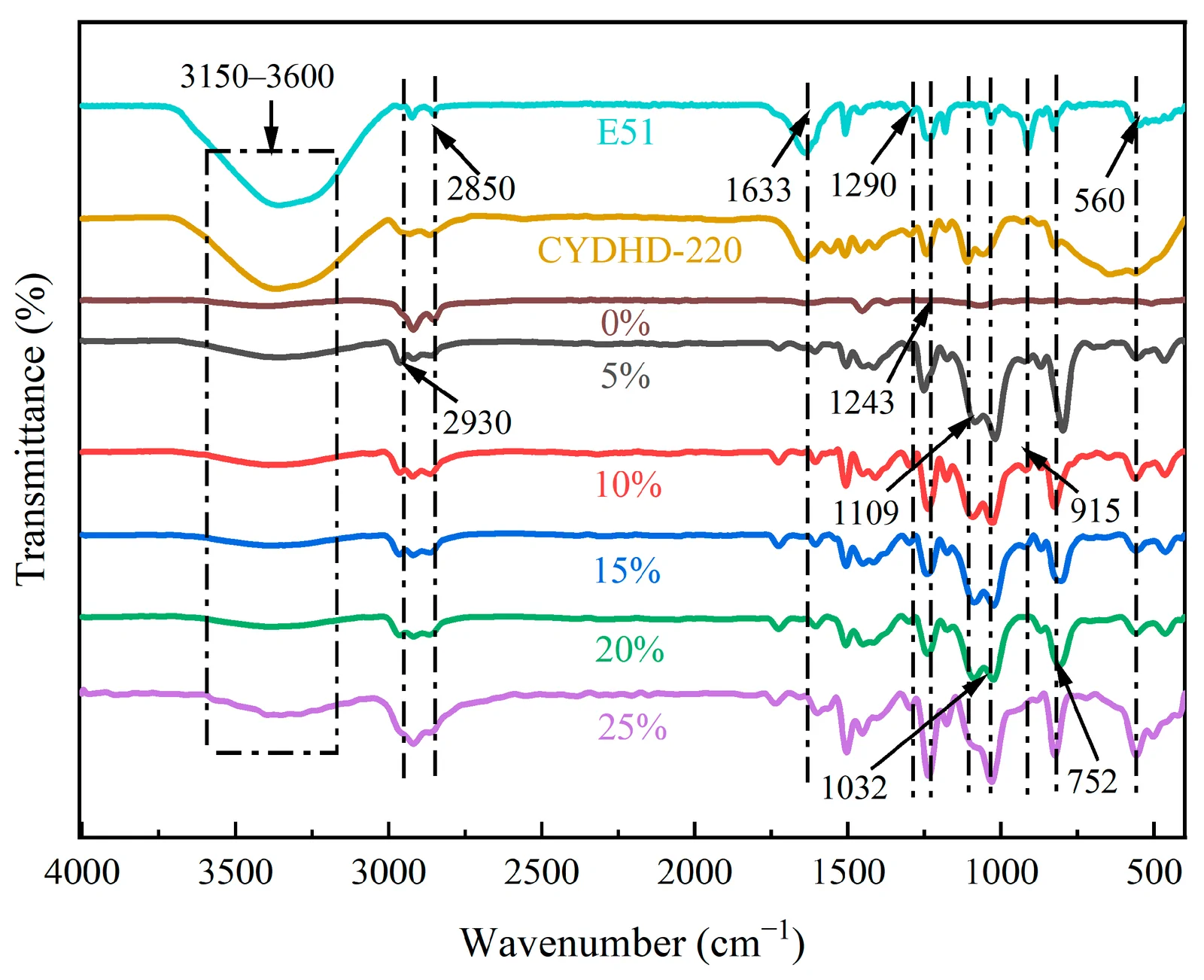
The FTIR test results of S-WEREA with different dosages, E51 resin, and curing agent CYDHD-220.

The FTIR test results of S-WEREA with different dosages, E51 resin, and curing agent CYDHD220 are shown in Figure 8. According to the wavenumbers 3150–3600, 2930, 2850, 752, and 560 cm^−1^, it could be observed that the FTIR spectrum of the S-WEREA was not a simple superimposition of WER and EA spectra, indicating that a chemical reaction had occurred in WER system. At the wavenumbers 3150–3600 cm^−1^, significant changes were observed in the composite stretching vibrations of C–H, N–H, and O–H bonds, suggesting that a strong chemical reaction between epoxy resin and curing agent occurred. As the epoxy functional groups were opened, the reaction products formed a large number of cured crosslinked structures and new functional groups. Obviously, in WEREA, the peaks of ether bonds (C–O–C) at wavenumbers 1243, 1109, and 1032 cm^−1^ and the peak of amine (C–N) at 1290 cm^−1^ disappeared, further manifesting that the reactions between amine, hydroxyl, and epoxy groups had substantially consumed. At wavenumbers 1633 cm^−1^, an absorption peak corresponding to the stretching vibration of the amide group (N–C=O) appeared in WEREA, which was absent in both the waterborne epoxy resin and the curing agent. Moreover, the peak at 915 cm^−1^, representing the epoxy chemical bond, did not appear in the WEREA. All the results demonstrated that the epoxide band at 915 cm^−1^ became undetectable within the FTIR detection limit, indicating significant consumption of epoxide groups.

### 3.7. DSR Analysis

#### 3.7.1. Frequency Sweep

(1) Han curve

Han curve, storage modulus (*G^′^*) versus loss modulus (*G^″^*) in logarithmic scale, was presented by Han based on the Boltzmann superposition principle [28]. It was concluded that for multiphase polymers, the relationship between storage modulus (*G^′^*) versus loss modulus (*G^″^*) could be given by the following equation, as shown in Equation (7):

(7) $$\mathrm{lg}G^{\prime }\left(\omega \right)=k\mathrm{lg}G^{\prime \prime }\left(\omega \right)+\left(1-k\right)\mathrm{lg}\left(\frac{8G_{N}^{0}}{\pi^{2}}\right)$$

where *ω* represents the frequency, *G^′^*(*ω*) is the storage modulus, *G^″^*(*ω*) is the loss modulus, $G_{N}^{0}$ is the plateau modulus, and k is the slope of the Han curve.

**Figure 9:**
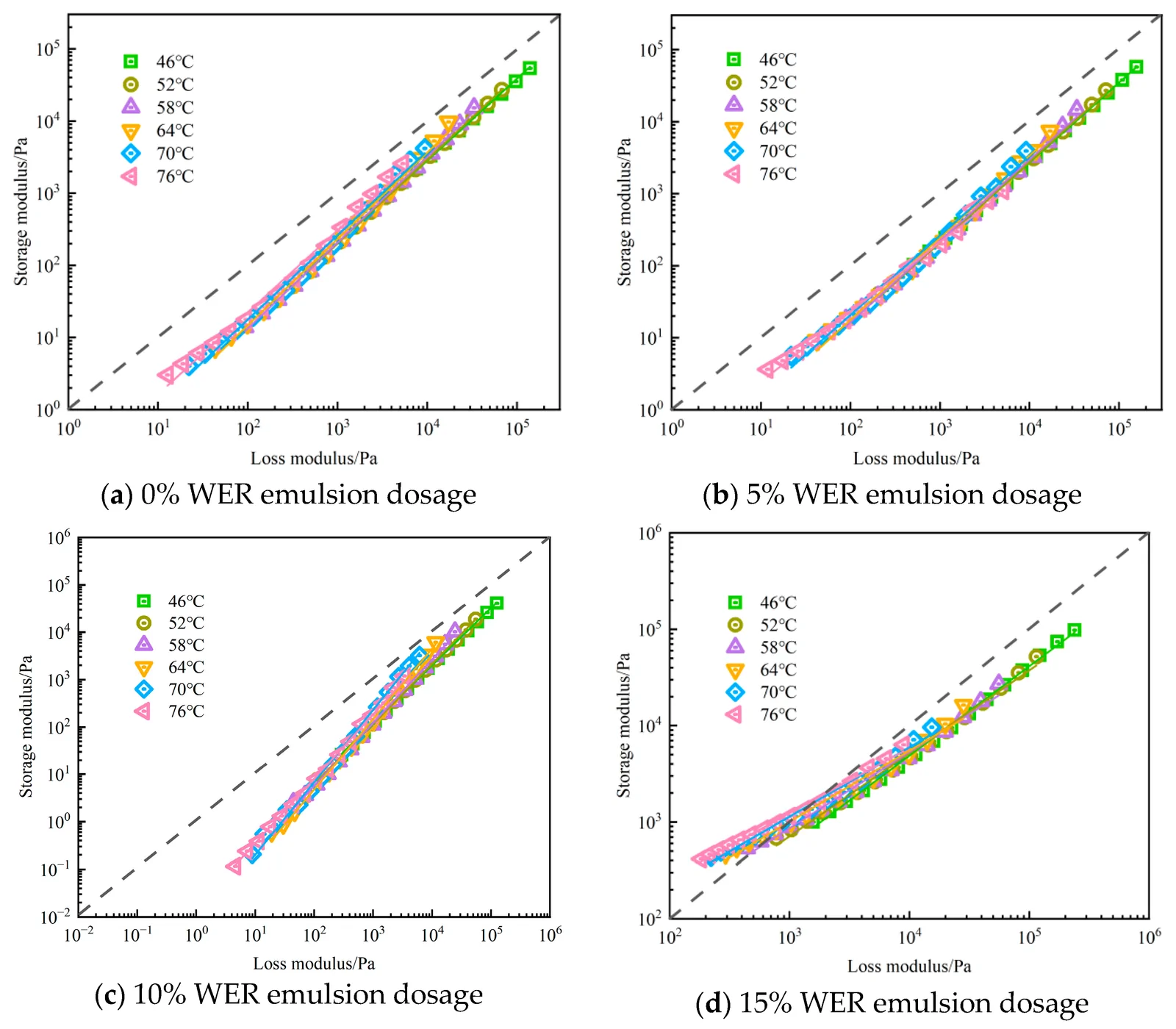

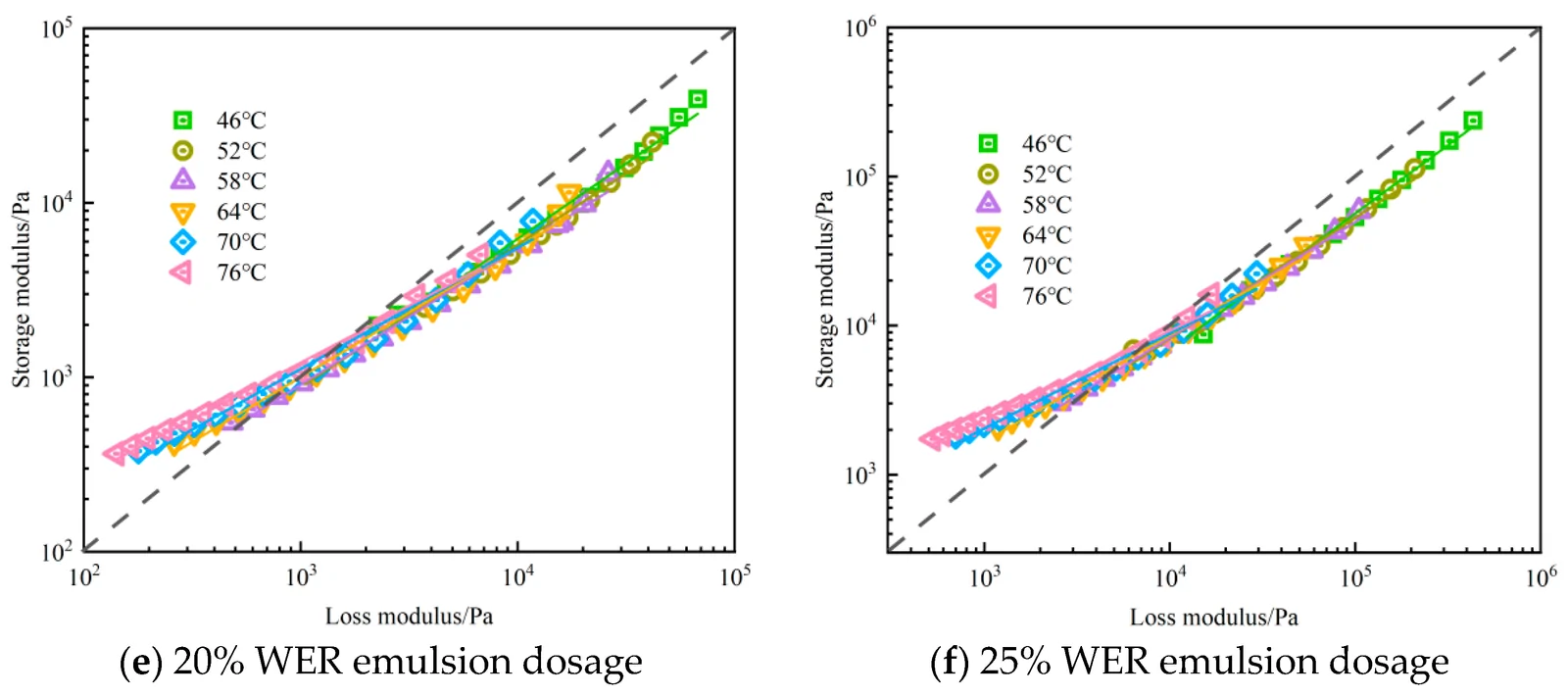
Han curve of varying WER emulsion dosage emulsified asphalt residue.

Generally, the closer the slope of the Han curve is to 2, the closer the multiphase polymer is to a homogeneous state. Based on the results of frequency sweeps, the viscoelastic behavior of the S-WEREA with varying WER content were studied by analyzing the changes in the slope of Han curve. The results of the fitting analysis were illustrated in Figure 9 and Table 7. It could be seen from Table 7 that the fitted R^2^ values of the Han curve for all samples were above 0.98, indicating the excellent logarithmic correlation between storage modulus (*G^′^*) and loss modulus (*G^″^*). The addition of WER had a significant impact on the Han curve of the samples. Specifically, the slope of curve for the demulsified asphalt without WER ranged from 1.21 to 1.53, whereas the slope for the S-WEREA containing 25% WER varied from 0.91 to 0.59.

While the dosages of WER were 5% and 10%, the cured product of WER in the S-WEREA could not form a network structure. Hence, the curves at different temperatures showed good overlap and also the changes in slopes were minimal, indicating relatively good homogeneity of the S-WEREA. However, when the dosage reached 15% or higher, the slope decreased rapidly with the increase in temperature and the S-WEREA showed the trend of phase separation. This could be attributed to the fact that the cured product of WER exhibited the remarkable thermodynamic differences from asphalt due to its thermosetting property. At room temperature, asphalt possessed higher viscosity and the cured product of WER could play a good role in preventing macroscopic phase separation. Nevertheless, as the temperature rose, the viscosity of asphalt decreased significantly while the thermodynamics behavior of the WER cured product was less affected by temperature, eventually leading to macroscopic phase separation.

(2) Master curve

According to the time-temperature superposition principle (TTSP), based on a reference temperature, the complex modulus (G^*^) and phase angle (*δ*) of S-WEREA under various temperatures and frequencies could be shifted to construct the master curve, respectively. The correlation of the shift from other temperatures to the reference temperature is referred to as the shift factor (*α_T_*), which could be calculated using the Williams Landel Ferry (WLF) equation, as shown in Equation (8) [29]. Zeng and Huang, using the CA model, proposed the modified Christensen–Anderson–Marasteanu (CAM) model, which more accurately described the viscoelasticity of asphalt materials. The master curve expressions using the modified CAM model are shown in Equations (9)–(11) [30].

(8) $$\mathrm{lg}\alpha_{T}=\frac{-C_{1}\left(T-T_{0}\right)}{C_{2}+\left(T-T_{0}\right)}$$

*f^′^* = *f* × *α_T_*(*T*) (9)

(10) $$G^{*}=G_{e}^{*}+\frac{G_{g}^{*}-G_{e}^{*}}{{\left[1+{\left(\frac{f_{c}}{f^{\prime }}\right)}^{k}\right]}^{\frac{m_{e}}{k}}}$$

(11) $$\delta =90I-\left(90I-\delta_{m}\right){\left\{1+{\left[\frac{\mathrm{lg}\left(\frac{f_{d}}{f^{\prime }}\right)}{R_{d}}\right]}^{2}\right\}}^{\frac{m_{d}}{2}}$$

where *T* is actual loading temperature, °C; *T*_0_ is reference temperature, °C; *C*_1_ and *C*_2_ are equations fitting temperature sensitivity parameters; *f^′^* is converted frequency; $G_{e}^{*}$ is the complex modulus at the equilibrium state (f→0), Pa; $G_{g}^{*}$ is the complex modulus at glass state (f→∞), Pa; *f_c_* is the location parameter with dimensions of frequency, Hz; *m_e_* and *k* are shape parameters; *δ_m_* is the max phase angle; *f_d_* is the position frequency, Hz; *R_d_* and *m_d_* are additional shape parameters; and *I* is an adjustment parameter.

**Figure 10:**
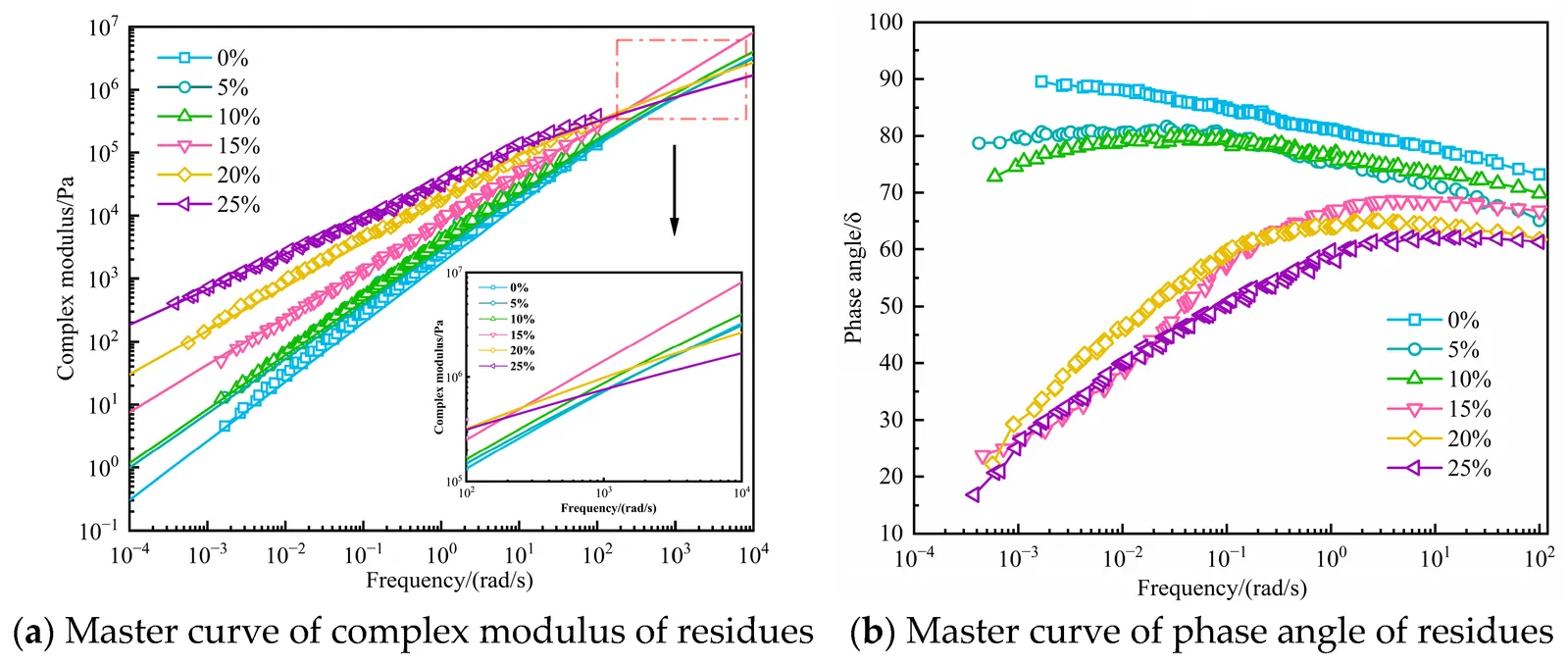
The master curve of the S-WEREA.

Based on the reference temperature of 46 °C, the shift factors were calculated by the WLF equation and data were fitted according to the CAM model. The master curve of the S-WEREA is shown in Figure 10.

From Figure 10a, it could be observed that the complex modulus of the S-WEREA rose with the increase in frequency and the content of WER. Within the low-frequency range, the differences in the complex modulus of the S-WEREA containing various contents of WER were significant, while these differences gradually diminished as the frequency increased. Moreover, the low-frequency region corresponds to high-temperature conditions in accordance with the TTSP, while the high-frequency region corresponds to low-temperature conditions. It could be seen that the addition of WER had a more pronounced effect on the high-temperature performance of the S-WEREA, while its impact on low-temperature performance was less. In addition, within the high-frequency range, the slope increments of the master curve decreased as the content of WER increased. According to the predicted trend of CAM model, the master curves of 20% and 25% WER intersected with each other, indicating that excessive amounts of WER could adversely affect the low-temperature performance of the S-WEREA. It should be emphasized that the low-temperature behavior is inferred from the DSR master curves via the time-temperature superposition principle (TTSP), rather than measured directly, since no direct low-temperature test (e.g., the bending beam rheometer test) or fracture test was performed in this study. DSR-based methods for evaluating the low-temperature behavior of asphalt binders have been reported in previous studies [31,32,33].

As shown in Figure 10b, the phase angle of the demulsified asphalt without WER decreased with increasing frequency, while the phase angle of the S-WEREA shown initial increase followed by a decrease and the phase angle of the former was always higher than that of the latter. Moreover, the phase angle of the S-WEREA exhibited slower variation within a certain frequency range, forming a “ plateau zone,” indicating that the S-WEREA maintained a stabler viscoelastic state. It was ascribed to the fact that the cured product of WER belonged to a thermosetting material and possessed greater elasticity than asphalt at higher temperatures, suggesting that the higher WER content, the more pronounced the elastic behavior of the S-WEREA.

Based on the master curve analysis of the complex modulus and phase angle, WER could significantly enhance the high-temperature performance of WEREA. However, at the 20% and 25% contents, WER had a negative influence on the low-temperature performance of WEREA. Moreover, at 5% and 10% contents, the improving effect of WER on the high-temperature performance was not remarkable. At a 15% content, the S-WEREA possessed the favorable low-temperature performance compared with 20% and 25% contents, providing a reference for the determination of WER content.

#### 3.7.2. MSCR

**Figure 11:**
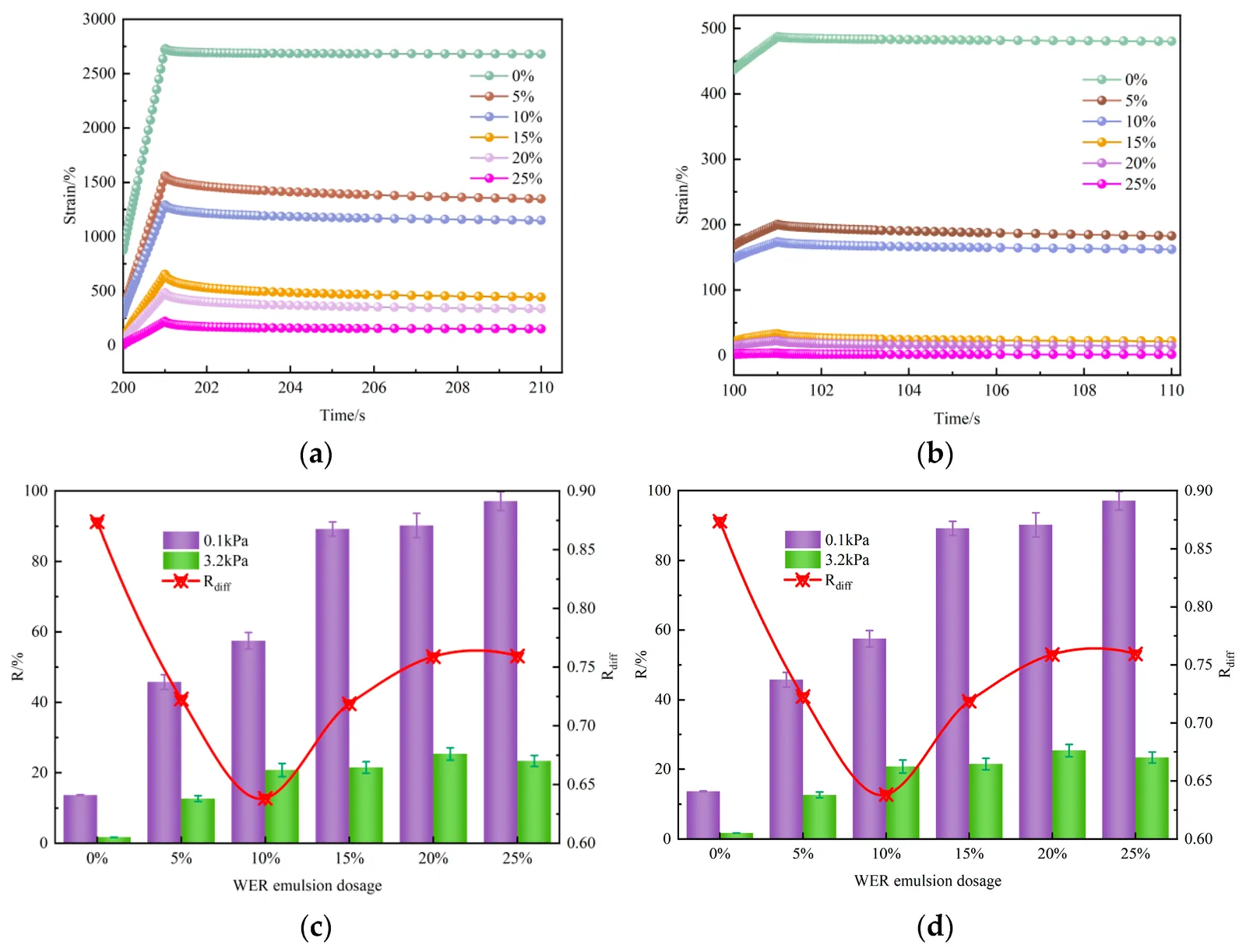
The creep recovery curve of residue under different stress levels during the first cycle (**a**,**b**); MSCR test results of evaporated residues (**c**,**d**). (**a**) Creep recovery curve of the first cycle of residue under 0.1 kPa; (**b**) creep recovery curve of the first cycle of residue under 3.2 kPa; (**c**) elastic recovery rate and its sensitivity of residues; (**d**) non-recoverable creep compliance and its sensitivity of residue.

It was well understood that the results of MSCR test could better reflect the rutting performance of polymer-modified asphalt than the rutting parameter G*/sin(δ) [34]. Figure 11a,b demonstrated the creep and recovery processes during the first cycle at 64 °C under the stress levels of 0.1 kPa and 3.2 kPa, respectively. The evaluation indices obtained from the test results included elastic recovery rate (*R*), non-recoverable creep compliance (*J_nr_*), relative difference in elastic recovery rate (*R_diff_*), and relative difference in non-recoverable creep compliance (*J_nrdiff_*), which were calculated using Equations (12)–(15).

(12) $$R=\frac{\varepsilon_{p}-\varepsilon_{u}}{\varepsilon_{p}-\varepsilon_{0}}\times 100\%$$

(13) $$J_{\mathrm{nr}}=\frac{\varepsilon_{u}}{\sigma }$$

(14) $$R_{\mathrm{diff}}=\frac{R_{0.1}-R_{3.2}}{R_{0.1}}\times 100\%$$

(15) $$J_{\mathrm{nrdiff}}=\frac{J_{\mathrm{nr}3.2}-J_{\mathrm{nr}0.1}}{J_{\mathrm{nr}0.1}}\times 100\%$$

where *ε_p_* represents the peak strain in the cycle, *ε_u_* denotes the residual strain in the cycle, and *ε*_0_ is the initial strain within the cycle; *σ* indicates the stress level in the cycle. *R*_0.1_ and *R*_3.2_ correspond to the elastic recovery rates at stress levels of 0.1 kPa and 3.2 kPa, respectively, while *J_nr_*_0.1_ and *J_nr_*_3.2_ represent the non-recoverable creep compliance at 0.1 kPa and 3.2 kPa stress levels, respectively.

As shown in Figure 11c,d, under the loads of 0.1 kPa or 3.2 kPa, the peak strain of the specimen was significantly affected by the content of WER. The higher the WER content, the lower the peak strain. R was defined as the ratio of recoverable strain to the total strain within a cycle, reflecting the high-temperature non-deformability of specimen. At the stress level of 0.1 kPa, when the WER content ranged from 0% to 15%, the R value exhibited a rapidly increasing trend. Meanwhile, when the WER content was from 15% to 25%, the R value only increased by 7.9%. At the stress level of 3.2 kPa, the R value of the S-WEREA remained notably higher than that of the demulsified asphalt without WER, indicating that the cured product of WER significantly enhanced the elastic recovery ability of WEREA.

*J_nr_* characterizes the permanent deformation of asphalt under repeated loading and is closely correlated with the rutting resistance of asphalt mixture [35]. At the stress level of 0.1 kPa, the *J_nr_* values of all specimens significantly decreased with the increase in WER content. Nevertheless, at the stress level of 3.2 kPa, the reduction in *J_nr_* value was less influenced by the increase in WER content. However, when the WER content exceeded 15%, the specimens exhibited strong stress sensitivity, resulting in a rapid increase in the *J_nrdiff_* value. Therefore, the excessive WER would bring the risk of WEREA failure when they were subjected to unexpected heavy traffic load or abnormal temperature rise in practical applications.

In summary, as the dosage of WER increased, the elastic recovery rate of the specimens rose, while the non-recoverable creep compliance decreases. This was attributed to the fact that the crosslinked network of the WER cured product enhanced the high-temperature deformation resistance of the S-WEREA. However, an excessive amount may lead to the hardening of the S-WEREA and a reduction in its toughness.

#### 3.7.3. LAS

**Figure 12:**
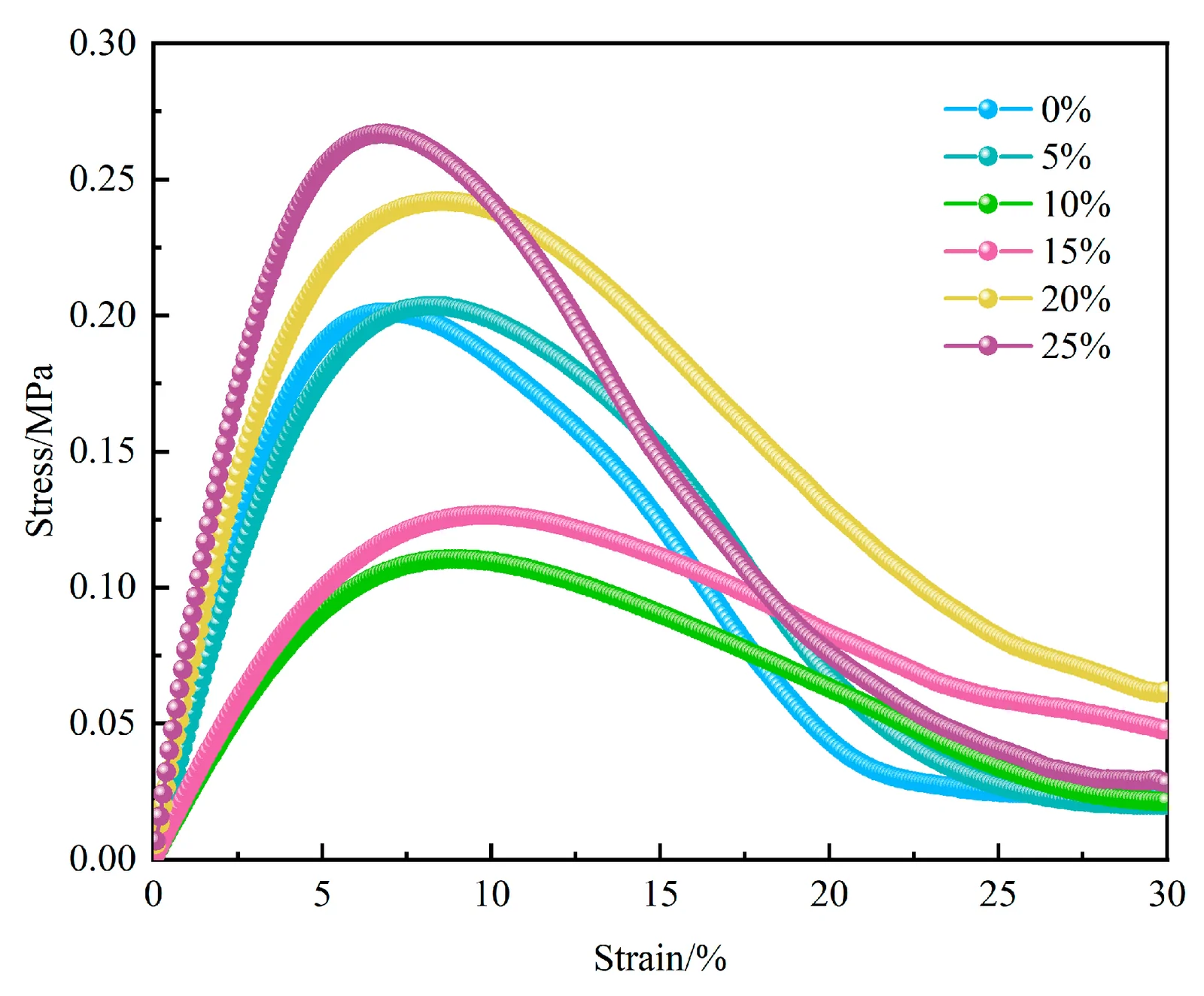
The stress–strain curve of the S-WEREA.

The stress–strain curves of the S-WEREA with six different WER contents were illustrated in Figure 12. As the specimens underwent fatigue failure, it could be observed that the stress first increased and then decreased as the strain grew. Similarly, while the WER content ranged from 0% to 25%, the failure strains corresponding to peak stresses, in turn, exhibited 6.80%, 8.16%, 8.84%, 9.91%, 8.55%, and 6.70%. Nevertheless, whether the higher content (20% and 25%) or the lower content (0% and 5%), the stress rose sharply with increasing strain, compared with medium content (10% and 15%). Especially, at 15% content, the S-WEREA showed higher failure strain and lower stress sensitivity, indicating better fatigue performance of the specimen.

Based on the results of frequency sweep and amplitude sweep tests, the cumulative fatigue damage, denoted as D, could be calculated by Equation (16) The parameter α, representing the undamaged performance of asphalt, could be determined from the test results of frequency sweep by Equations (17) and (18). The relationship between the loss modulus and the cumulative fatigue damage variable was expressed in Equation (19), while the fatigue failure criterion was given in Equation (20), through which the fatigue life could be predicted.

(16) $$D(t)≅\sum_{i=1}^{N}{\left[\pi I_{D}\gamma_{0}^{2}(\left|G^{*}\right|\sin\delta_{i-1}-G^{*}\left|\sin\delta_{i}\right|)\right]}^{\frac{\alpha }{1+\alpha }}{\left(t_{i}-t_{i-1}\right)}^{\frac{1}{1+\alpha }}$$

log*G^′^*(*ω*) = *m*(log*ω*) + *b* (17)

(18) $$\alpha =1+\frac{1}{m}$$

(19) $$C\left(t\right)=C_{0}-C_{1}{\left(D\right)}^{C_{2}}$$

(20) Df=0.35C0C11c2

where *I_D_* represents the complex modulus at 1% strain level, MPa; *γ*_0_ denotes the strain level, %; |G^*^| is the complex modulus, MPa; *t_i_* represents the test time, s; *G^′^*(*ω*) is storage modulus, MPa; *ω* stands for the frequency, Hz; *m* is the fitting parameter; *C*_0_ is assigned the value of 1; and *C*_1_ is shape parameters of the fitting function determined through optimization.

**Figure A5:**
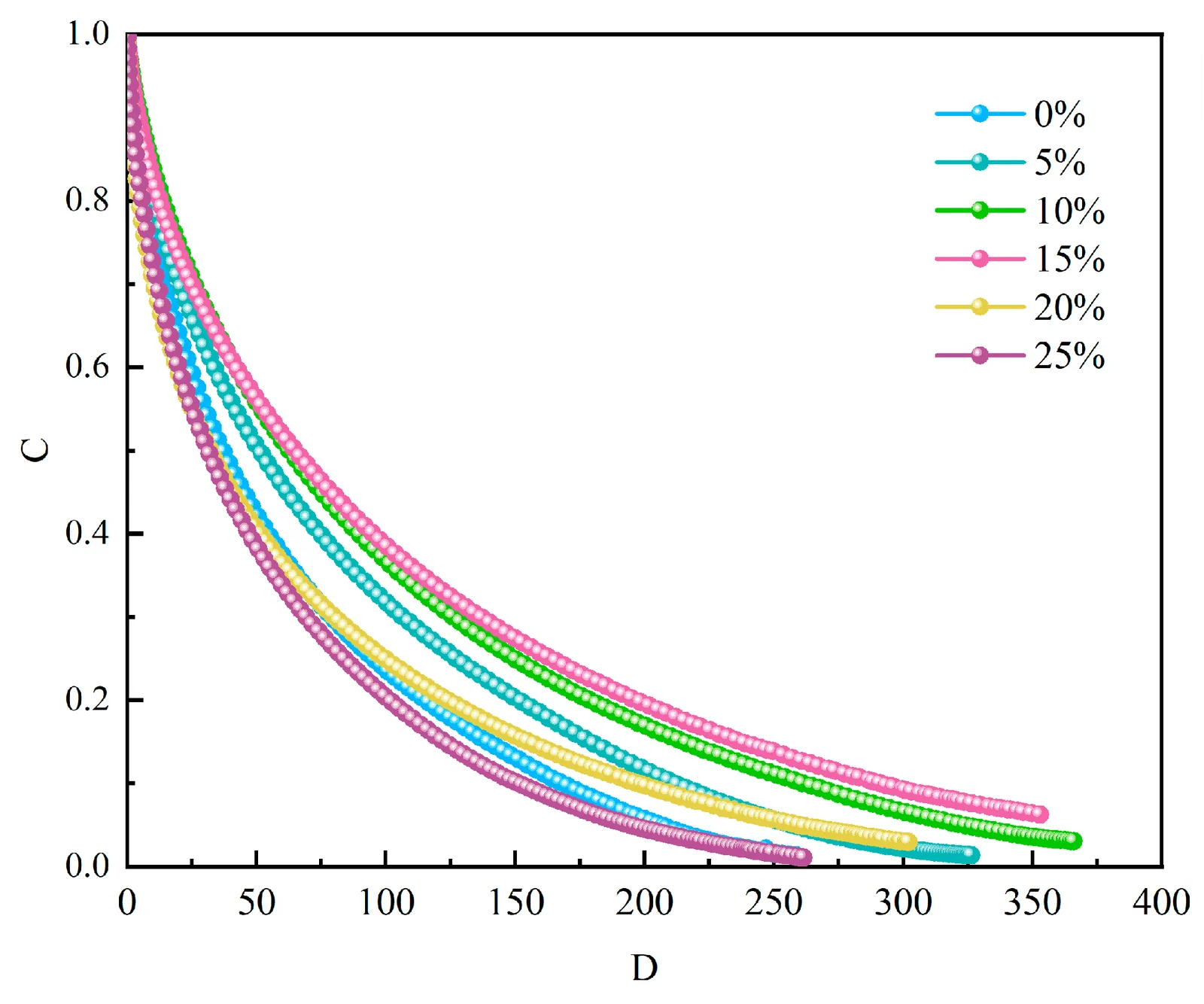
The fatigue damage curve of residue.

In accordance with the viscoelastic continuum damage (VECD) model, the relationship between fatigue life and strain was established, while the fatigue damage curve was demonstrated in Figure A5, where the vertical axis C and the horizontal axis D represented the integrity parameter and cumulative damage parameter, respectively. When the D value was the same, the specimen with higher C value possessed better fatigue resistance [36]. The values of C at any position with same D presented in the order: 15% > 10% > 5% > 20% > 0% > 25%. Hence, the 15% WER content contributed to enhancing the fatigue resistance of WEREA.

Additionally, the parameters derived from the viscoelastic continuum damage model and the predicted fatigue lives were presented in Table 8. The fatigue life, *N_f_*, was calculated by Equations (21)–(23), and 2.5% *N_f_* and 5% *N_f_* represented the fatigue life at strain of 2.5% and 5%, respectively.

*k* = 1 + (1 − *C*_2_)*α* (21)

(22) $$A_{35}=\frac{f{\left(D_{f}\right)}^{k}}{k{\left(\pi I_{D}C_{1}C_{2}\right)}^{\alpha }}$$

(23) $$N_{f}=A_{35}{\left(\gamma_{\max}\right)}^{B}$$

where *C*_2_ is shape parameters of the fitting function determined through optimization; *f* is the loading frequency of 10 Hz; A35 is the fatigue parameter calculated when the fatigue factor decreases to 35% of its initial value; *γ_max_* is the estimated maximum strain of the pavement structure, %; and *B* is the strain sensitivity parameter, equal to −2*α*.

**Figure 13:**
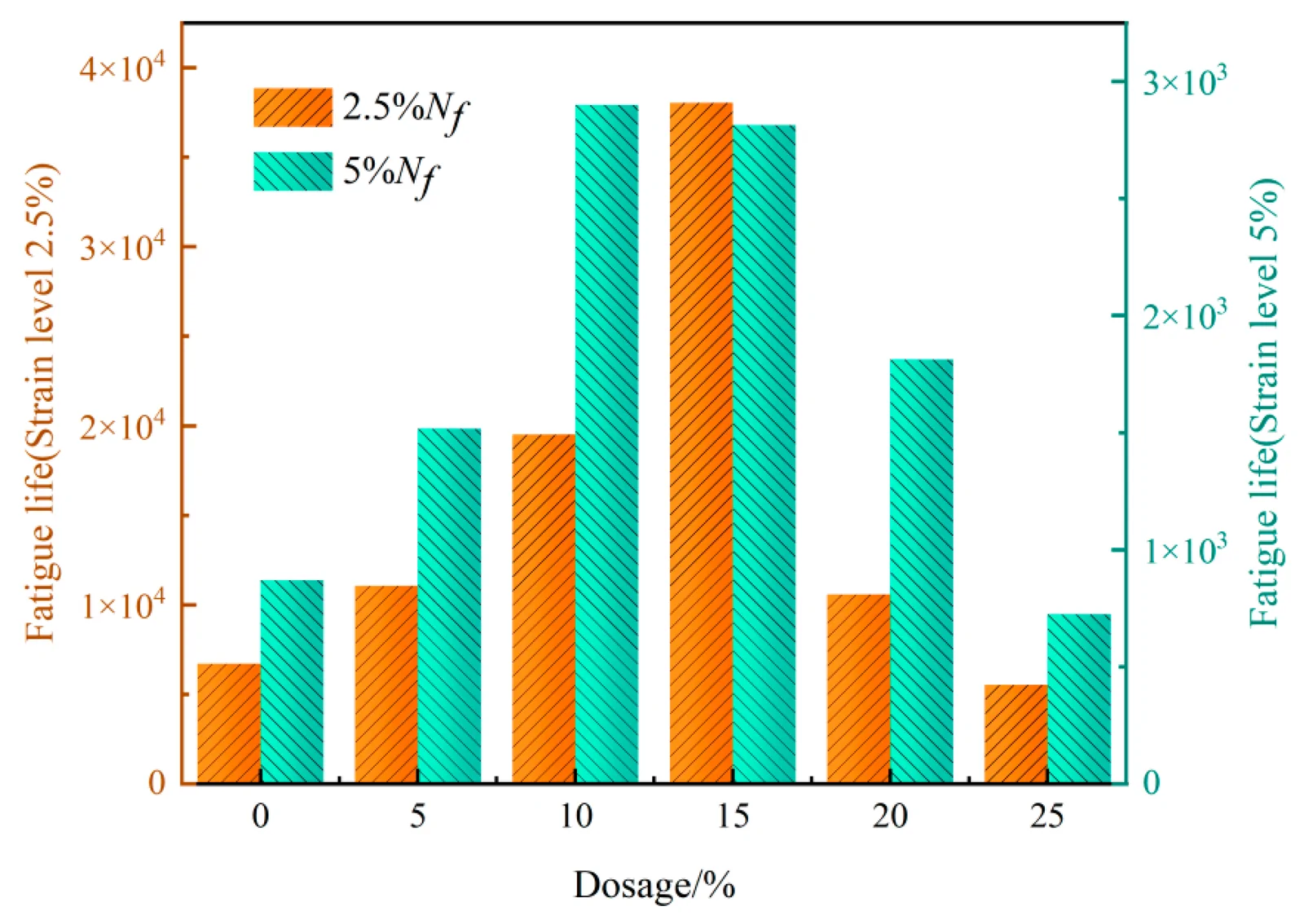
Fatigue life at strain levels of 2.5% and 5%.

According to the study of Prabin Kumar Ashish, it was reasonable that the fatigue life of high-grade asphalt pavement was predicted at strain level of 2.5%, while one of the ordinary asphalt pavements was more appropriate for being predicted at a strain level of 5.0% [36]. Based on the calculations, the 15% content of WER could be more suitable for high grade asphalt pavement, whereas the 10% content could be more appropriate for ordinary asphalt pavements (Figure 13). Fatigue performance reflected the serviceability of asphalt binder under long-term repeated traffic loads. Excessive WER could result in uneven phase structure of the S-WEREA, making fatigue failure be more likely to be initiated at the phase interface. A reasonable amount of WER should facilitate the phase structure of the S-WEREA being more homogeneous and thereby enhance its fatigue resistance.

In summary, the WER content of 15% was determined solely from the changes in the rheological properties of S-WEREA with WER content. This value is a content favorable for the rheological behavior of the binder under study, and the optimal WER content for micro-surfacing mixtures should be verified in subsequent studies.

## 4. Conclusions

This work focuses on developing the emulsifier formulation, the effects of the emulsifier and preparation method on WER particle size, and curing behavior. Based on the experimental results, the following main conclusions are drawn:

1. The optimal preparation parameters for WER were determined as follows: a composite emulsifier ratio of SDS:Span-80 = 5:13 (HLB value 14.2), an emulsifier dosage of 12%, a shear rate of 4712 s^−1^, and a shear temperature of 60 °C. Under these conditions, WER exhibited minimal particle size, a narrow size distribution, and optimal emulsification performance.
2. With the increase in WER content, the viscosity of WEREA gradually rose. Specifically speaking, initially rising slowly, and then rapidly increasing due to curing reactions between epoxy resin and curing agent. Ultimately, the viscosity of WEREA gradually approached a stable value as the curing reaction proceeded.
3. As the amount of WER increased, the fluorescent area fraction gradually increased, and the cured products of WER were qualitatively observed to change from dispersed “sea-island” structures into a continuous network. This morphological evolution is consistent with the improved high-temperature rheological response indicated by the DSR results, although a quantitative correlation between the morphology and the rheological indices was not established.
4. Based on the change in the rheological properties of S-WEREA with WER content, a 15% WER content gave the most favorable balance of high-temperature stability and fatigue resistance; this value is a preliminary rheological compromise, and the optimal WER content for micro-surfacing mixtures should be verified in subsequent studies. In addition, excessive WER may adversely impact the low-temperature performance of S-WEREA and also lead to macroscopic phase separation at high-temperature. It should be emphasized that no direct low-temperature (BBR) or fracture test was performed in this study; consequently, the conclusions regarding low-temperature performance rest solely on rheological inferences derived from the master curves.

## Figures and Tables

**Table 1:** Properties of SBR modified emulsified asphalt.

Tested Property		Units	Value	Standard
Residue on sieve		%	0.02	AASHTO T59-22 [14]
Content of evaporation residue		%	60	
Storage stability	1d	%	0.3	AASHTO T59-22
	5d		1.1	
Properties of evaporation residue	Penetration (25 °C)	0.1 mm	58	AASHTO T49-22 [15]
	Softening point	°C	55.4	AASHTO T53-22 [16]
	Ductility (5 °C)	cm	120	AASHTO T301-22 [17]

**Table 2:** Properties of epoxy resin.

Type of Epoxy Resin	Tested Property	Units	Value
E-51	Epoxy content	g/mol	184–195
	Hydrolyzed chlorine	%	≤0.20
	Inorganic chlorine	mg/kg	≤50
	Viscosity in 25 °C	mPa∙s	10,000–16,000

**Table 3:** Properties of curing agent.

Type	Tested Property	Units	Value
CYDHD-220	Appearance	-	Yellow
	Viscosity (25 °C)	mPa∙s	300–2000
	Active hydrogen equivalent	%	200–240

**Table 4:** Properties of emulsifiers.

Type	Tested Property	Units	Value
SDS	Density	%	1.022
	ph	-	8–11
	State	-	Particles
Span-80	Density	%	0.986
	ph	-	5–8
	State	-	Liquid

**Table 5:** Design of composite emulsifiers.

SDS:Span-80 (Mass Ratio)	HLB Values of Composite Emulsifiers
1:2	16.2
1:5	10.3
1:7	8.8
2:7	12.2
5:8	18.0
5:13	14.2

**Table 6:** Area percentage of the WER cured product in binarized images.

Dosage/%	*i*	*j*	R/%
0	0	11,999,857	0
5	383,995	11,999,857	3.2
10	995,988	11,999,857	8.3
15	1,655,980	11,999,857	13.8
20	2,087,975	11,999,857	17.4
25	2,639,384	11,999,857	22.0

**Table 7:** Linear fitting results of the Han curve.

	Temperature /°C	46	52	58	64	70	76
Dosage/%							
0	intercept	−1.0984	−1.1905	−1.2588	−1.2285	−1.0904	−0.9260
	slope	1.2137	1.2685	1.3364	1.4867	1.5290	1.3602
	R^2^	0.9994	0.9999	0.9999	0.9952	0.9937	0.9905
5	intercept	−1.0041	−1.0385	−1.0476	−1.0289	−0.8870	−0.5774
	slope	1.1373	1.1662	1.1912	1.1977	1.1687	1.1372
	R^2^	0.9999	0.9995	0.9995	0.9907	0.9881	0.9949
10	intercept	−1.5574	−1.7440	−1.9223	−2.2945	−2.2263	−1.8400
	slope	1.1088	1.1206	1.1300	1.1391	1.1019	0.9683
	R^2^	0.9997	0.9998	0.9987	0.9959	0.9934	0.9949
15	intercept	−0.0267	0.2702	0.5699	0.7887	0.8996	1.0610
	slope	0.9286	0.8606	0.7888	0.7380	0.7189	0.6764
	R^2^	0.9976	0.9934	0.9894	0.9849	0.9799	0.9887
20	intercept	0.3414	0.4935	0.6139	0.7716	0.9605	1.1276
	slope	0.8631	0.8166	0.7810	0.7430	0.6956	0.5933
	R^2^	0.9897	0.9944	0.9936	0.9848	0.9827	0.9887
25	intercept	0.1808	0.6632	0.8160	1.0802	1.4004	1.5914
	slope	0.9143	0.8119	0.7757	0.7140	0.6365	0.5933
	R^2^	0.9955	0.9912	0.9967	0.9921	0.9838	0.9797

**Table 8:** LAS test results for residues.

Dosage/%	α	A_35_	B	C_1_	C_2_	2.5% *N_f_*	5% *N_f_*
0	1.475	1.002 × 10^5^	−2.949	0.119	0.390	6716	870
5	1.433	1.530 × 10^5^	−2.867	0.087	0.432	11,064	1517
10	1.375	2.424 × 10^5^	−2.750	0.072	0.450	19,514	2901
15	1.878	1.189 × 10^6^	−3.757	0.162	0.322	38,040	2814
20	1.273	1.092 × 10^5^	−2.545	0.068	0.466	10,583	1813
25	1.467	8.128 × 10^4^	−2.933	0.149	0.351	5529	724

**Figure A3:**
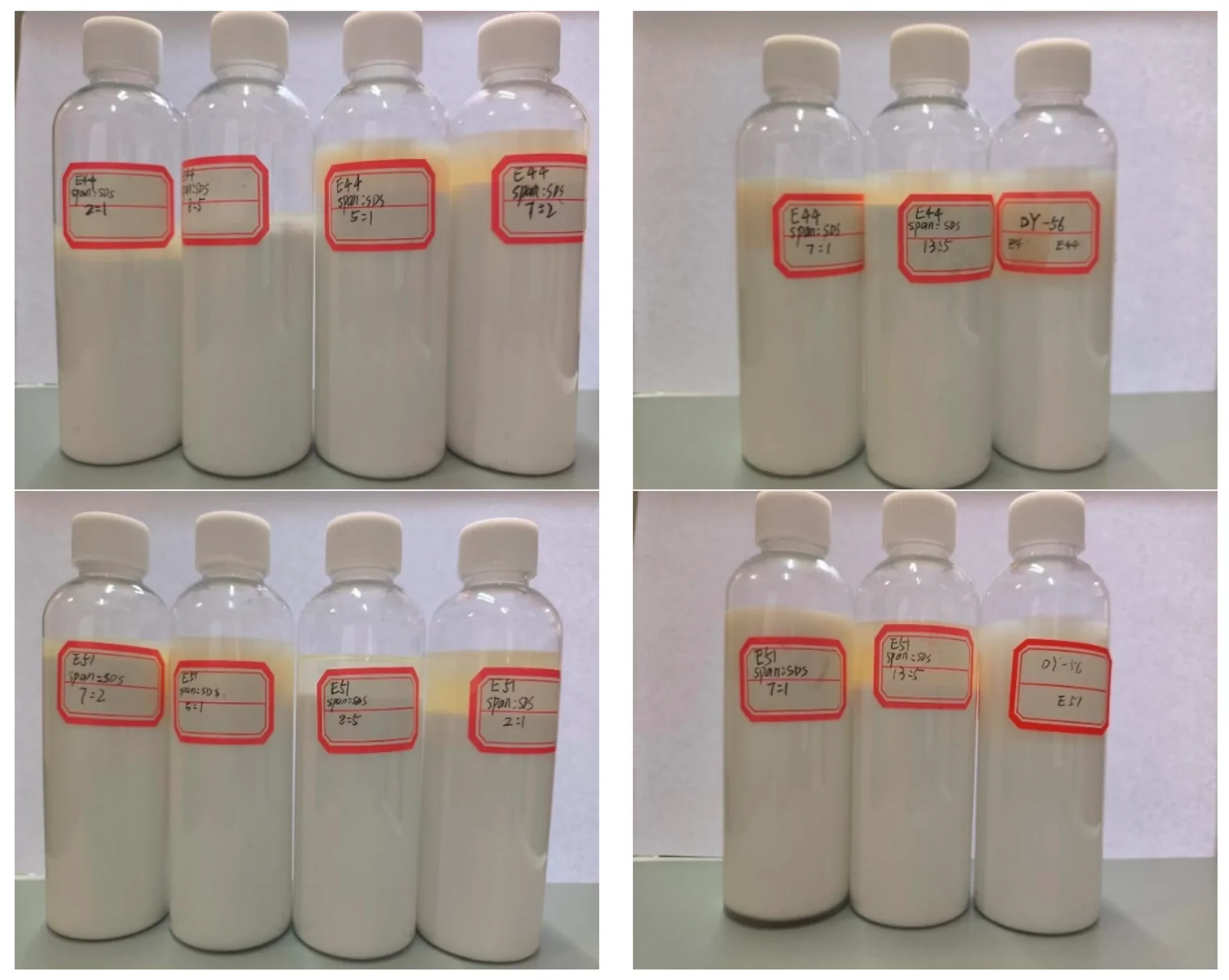
The visual appearance of the WER emulsions during storage. (A simple sedimentation observation was conducted at room temperature for 1, 7, 14 and 60 days, with three parallel samples in each group.)

## Data Availability

The original contributions presented in this study are included in the article. Further inquiries can be directed to the corresponding author.

## Abbreviations

The following abbreviations are used in this manuscript:

WER	Waterborne epoxy resin
EA	Emulsified asphalt
WEREA	Waterborne epoxy resin emulsified asphalt
S-WEREA	Solidified waterborne epoxy resin emulsified asphalt
SDS	Sodium dodecyl sulfate
HLB	Hydrophilic–lipophilic balance
FTIR	Fourier-transform infrared spectroscopy
SBR	Styrene-butadiene rubber
LAS	Linear amplitude sweep
MSCR	Multiple stress creep and recovery
DSR	Dynamic shear rheometer
FM	Fluorescence microscopy
LVE	Linear viscoelastic region
TTSP	Time-temperature superposition principle
VECD	Viscoelastic continuum damage
ATR	Attenuated total reflection
IPP	Image ProPlus
RV	Rotational viscometry
PU	Polyurethane
SBS	Styrene-butadiene-styrene block copolymer
CA	Christensen–Anderson model
CAM	Christensen–Anderson–Marasteanu model

## Appendix A

(A1) $$\gamma =\frac{\mathrm{\pi Dn}}{60\delta }$$

where *γ* is the shear rates (s^−1^), *D* is the stirring-head diameter (m), *n* is the shear mixer rotational speeds (r/min), and *δ* is the rotor–stator clearance (m).
